# Pregnancy state before the onset of labor: a holistic mechanical perspective

**DOI:** 10.1007/s10237-024-01853-3

**Published:** 2024-05-17

**Authors:** Daniel S. Fidalgo, Renato M. Natal Jorge, Marco P. L. Parente, Erin M. Louwagie, Ewelina Malanowska, Kristin M. Myers, Dulce A. Oliveira

**Affiliations:** 1 Institute of Science and Innovation in Mechanical and Industrial Engineering (INEGI), R. Dr. Roberto Frias 400, 4200-465 Porto, Portugal; 2 Mechanical Department (DEMec), Faculty of Engineering of University of Porto (FEUP), R. Dr. Roberto Frias, 4200-465 Porto, Portugal; 3 Department of Mechanical Engineering, Columbia University, New York, NY 10027 USA; 4 Department of Gynaecology, Endocrinology and Gynaecologic Oncology, Pomeranian Medical University, Szczecin, Poland

**Keywords:** Cervical shortening, Cervical dilation, Cervical contractions, Fetal membrane rupture, Vaginal delivery, Numerical simulations

## Abstract

Successful pregnancy highly depends on the complex interaction between the uterine body, cervix, and fetal membrane. This interaction is synchronized, usually following a specific sequence in normal vaginal deliveries: (1) cervical ripening, (2) uterine contractions, and (3) rupture of fetal membrane. The complex interaction between the cervix, fetal membrane, and uterine contractions before the onset of labor is investigated using a complete third-trimester gravid model of the uterus, cervix, fetal membrane, and abdomen. Through a series of numerical simulations, we investigate the mechanical impact of (i) initial cervical shape, (ii) cervical stiffness, (iii) cervical contractions, and (iv) intrauterine pressure. The findings of this work reveal several key observations: (i) maximum principal stress values in the cervix decrease in more dilated, shorter, and softer cervices; (ii) reduced cervical stiffness produces increased cervical dilation, larger cervical opening, and decreased cervical length; (iii) the initial cervical shape impacts final cervical dimensions; (iv) cervical contractions increase the maximum principal stress values and change the stress distributions; (v) cervical contractions potentiate cervical shortening and dilation; (vi) larger intrauterine pressure (IUP) causes considerably larger stress values and cervical opening, larger dilation, and smaller cervical length; and (vii) the biaxial strength of the fetal membrane is only surpassed in the cases of the (1) shortest and most dilated initial cervical geometry and (2) larger IUP.

## Introduction

Successful parturition relies on synchronized interaction between three biological structures: the body of the uterus, the cervix, and the fetal membrane (Vink 2020). In normal vaginal deliveries, the following ordered sequence usually occurs: (1) cervical ripening, (2) uterine contractions, and (3) fetal membrane rupture (Vink and Feltovich 2016). A different sequence is verified when premature rupture of the fetal membrane or preterm labor happens, and it may include early cervical ripening (cervical insufficiency), preterm premature rupture of the fetal membrane, and preterm uterine contractions (Vink 2020). These may lead to preterm birth, whose global rates have increased from 9.8% in 2000 to 10.6% in 2014 (Vink 2020; Walani 2020). Surviving babies may suffer several short-term morbidities (De Araújo et al. 2012; Mwaniki et al. 2012; Platt 2014) and long-term difficulties (Teune et al. 2011; Vogel et al. 2018). There are currently no accurate methods to determine whether patients will undergo any of these complications, or any treatments to prevent them effectively (Vink 2020). Cervical cerclage, progesterone, antibiotic prophylaxis, and anti-inflammatory treatments are common procedures applied to prevent preterm birth, but none of them offers total efficiency for every woman (Flood and Malone 2012). A complete characterization of the biomechanisms by which women go into labor at full term is still missing. Since these phenomena are naturally mechanical, we need a better understanding of the uterine, cervical, and membrane mechanical response during birth (Voltolini et al. 2013).

The uterine body is a complex organ that comprises billions of smooth muscle cells able to generate large forces and facilitate birth (Rudolph and Ivy 1930). These cells transmit electrical signals along the uterine wall (action potentials). During a normal vaginal delivery, these impulses are synchronized, creating a propulsive force due to uterine contractions (Bhogal 2017; Young 2016). This process is accompanied by cervical dilation, resulting in the expulsion of the fetus from the uterine cavity (Bastos et al. 2012).

The cervix is at the base of the uterus and comprises cervical epithelial and stromal layers (Tantengco and Menon 2020). The cervix must remain closed to support the mechanical loads developed by the growing fetus throughout most of the gestation (Shi et al. 2019). The cervix softens throughout pregnancy, in preparation for labor (Badir et al. 2013), but if cervical softening occurs too soon, there is a risk of preterm birth (Vink 2020). Thus, the cervix undergoes an important remodeling process throughout gestation, involving rearrangement and realignment of collagen fibrils, and changes in the extracellular matrix (ECM) (Barnum et al. 2022; Myers et al. 2009; Nallasamy et al. 2021; Tantengco and Menon 2020; Yao et al. 2016).

It has been hypothesized that the cervix responds passively to uterine contractions during the onset of labor (Tantengco and Menon 2020). However, some studies have investigated the existence of active cervical contractions during gestation and parturition (Rudel and Pajntar 1999; Tantengco and Menon 2020). Smooth muscle fibers represent approximately 10% to 45% of the cervical stroma, besides collagen and ECM (Verdenik et al. 2001; Vink et al. 2016). These smooth muscle fibers may create an active response in the cervix and lead to cervical remodeling (Tantengco and Menon 2020). Other studies have identified longitudinal and circumferential collagen fibers in the cervix: The longitudinal fibers may contract and contribute to cervical dilation, while the circumferentially aligned fibers may keep the cervix closed (Tantengco and Menon 2020; Vink et al. 2016; Weiss et al. 2006; Yao et al. 2016).

The fetal membranes are layered biological structures surrounding and protecting the fetus throughout pregnancy, working as a mechanical and immunological barrier (Bryant-Greenwood 1998; Jabareen et al. 2009). The amnion and the chorion, whose microstructures differ significantly from each other, are the main layers of fetal membranes (Buerzle and Mazza 2013). The amnion is the thinner, stiffer, and stronger inner layer of the membrane facing the amniotic fluid, while the chorion is the more compliant and extensible outer layer contacting the uterine body (Buerzle et al. 2012; Buerzle and Mazza 2013; Ilancheran et al. 2009; Mauri et al. 2016b; Oyen et al. 2004; Zhang et al. 2021). A maternal layer called decidua shares a close anatomical structure with the chorion and results from the differentiation process of the uterine environment (Abbas et al. 2019; Malak and Bell 1994; Tahan and Tahan 2014).

In the late stages of pregnancy, an area of altered morphology occurs within the region of fetal membranes that overlies the cervix (Verbruggen et al. 2017). This region, known as the “weak zone,” is characterized by reduced strength. It has been hypothesized that fetal membrane rupture initiates in this area (Verbruggen et al. 2017).

Several efforts have been made to characterize the complex uterine environment during different stages of pregnancy. Vila Pouca et al. simulated uterine contractions to reproduce the initial moments of the second stage of labor (Vila Pouca et al. 2019). Fidalgo et al. studied the impact of irregular uterine contraction on labor progression (Fidalgo et al. 2022). Buerzle and Mazza et al. and Fidalgo et al. developed a mechanical constitutive model to characterize the fetal membrane response (Buerzle and Mazza 2013; Fidalgo et al. 2024). The novelty of this work resided in bringing all these aspects together in one numerical model to simulate the phenomena occurring moments before the onset of labor.

In this work, the complex interaction between the cervix, fetal membrane, and uterine contractions before the onset of labor was analyzed, more specifically, the impact of the following physiological conditions on the maximum principal stress distribution, cervical remodeling in terms of shape change, and fetal membrane rupture: (i) initial cervical shape, (ii) cervical stiffness, (iii) cervical contractions, and (iv) intrauterine pressure. A complete parametrized numerical model of the full-term gravid uterine environment was developed, comprising the cervix, body of the uterus, fetal membrane, and abdomen. Each structure was characterized by appropriate constitutive models previously developed and validated: The uterine body and cervix were modeled through an electro-chemo-mechanical model able to mimic a set of muscle contractions (Fernandez et al. 2016; Fidalgo et al. 2022); the amnion layer from the fetal membrane was characterized by a set of anisotropic constitutive equations following a membrane model formulation (Fidalgo et al. 2024); elastic linear models characterized the chorion and the decidua (Fidalgo et al. 2024); and the neo-Hookean constitutive model characterized the abdomen (Westervelt et al. 2017).

This is the first numerical study embracing a complete full-gravid uterine and cervical model, uterine and cervical contractions, and a diversity of features associated with pregnancy. We hope to gain more insight into what conditions may influence a successful vaginal delivery and clarify the complex biomechanical interaction between the cervix, fetal membrane, and body of the uterus, which is not entirely understood.

## Methods

The active and passive constitutive material models, finite element analysis setup, and numerical simulation parameters for a full-term gravid pregnancy biomechanical model are detailed in this section.

### Material constitutive models

The constitutive material models used to define the uterus, cervix, and amnion are described in subSects. 2.1.1 and 2.1.2. The remaining components of the parametrized full-term gravid uterine model (chorion, decidua, and abdomen) were defined by linear elastic or neo-Hookean constitutive models.

#### Uterine and cervical electro–chemo–mechanical constitutive model

Uterine contractions are triggered by electrical, chemical, and mechanical stimuli (Fidalgo et al. 2022; Sharifimajd et al. 2016; Sharifimajd and Stålhand 2014; Yochum et al. 2016). The complete model that was implemented to define uterine and cervical contractions is divided into three sub-models:

- Electric model—represents the ionic currents responsible for the smooth muscle cells' activity and the output is the ${[{{\text{Ca}}}^{2+}]}_{i}$ dynamics;
- Chemical model—determines the fraction of cross-bridges;
- Mechanical model—computes the passive and active responses of the muscle.

**Fig. 1:**
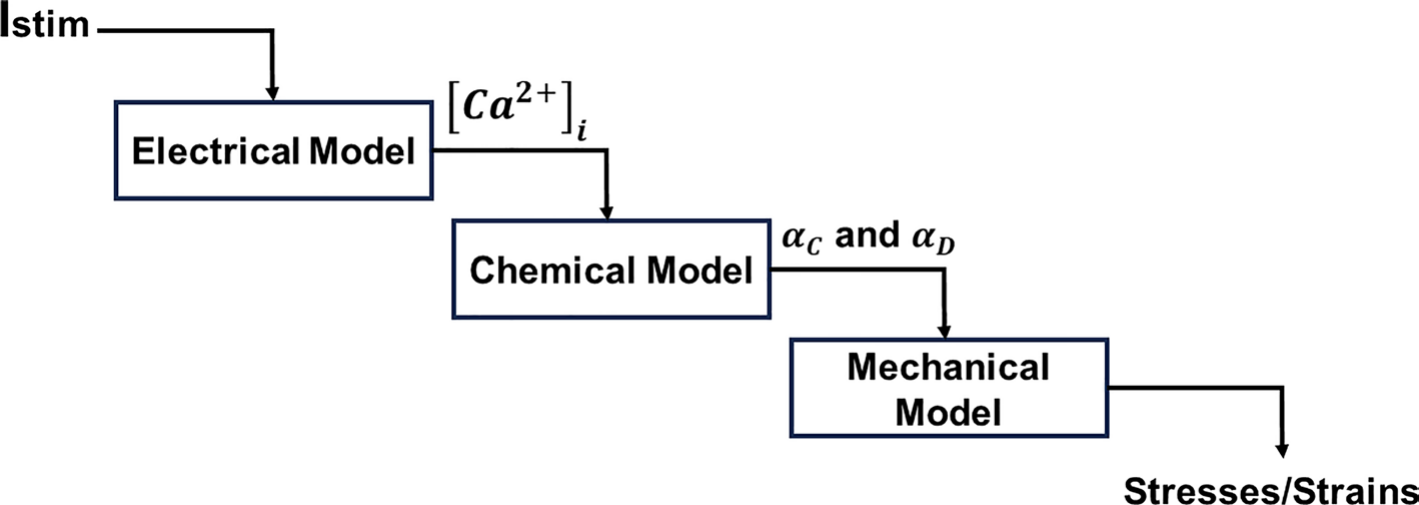
Representative scheme of the electro-chemo-mechanical model. ${I}_{{\text{stim}}}$ represents the stimulation current which will activate the electrical model and, consequently the entire constitutive model

The intracellular calcium concentration ${[{{\text{Ca}}}^{2+}]}_{i}$ links the electrical to the chemical sub-models. The link between the latter and the mechanical model is given by the fraction of cross-bridges ${\alpha }_{{\text{C}}}$ and ${\alpha }_{D}$, which determines the active response of the muscle (Fig. 1).

Due to the complexity of the electro-chemo-mechanical constitutive model, only the main equations and parameters are described here. A more detailed description of the model can be found at Fidalgo et al. (2022).

The intracellular calcium concentration ${[{{\text{Ca}}}^{2+}]}_{i}$ is determined through the following equation:

2.1 $$\frac{{d\left[ {{\text{Ca}}^{2 + } } \right]_{i} }}{{{\text{d}}t}} = f_{{\text{c}}} \left( { - AI_{{{\text{Ca}}}} - K_{{{\text{Ca}}}} \left[ {{\text{Ca}}^{2 + } } \right]_{i} } \right)$$

where ${f}_{{\text{c}}}$ represents the calcium influx probability, $A$ is the current conservation factor, ${I}_{{\text{Ca}}}$ corresponds to the ionic current for the voltage-dependent calcium channel, and ${K}_{{\text{Ca}}}$ is the calcium extraction factor.

${\alpha }_{{\text{C}}}$ and ${\alpha }_{D}$ represent the myosin in a bound phosphorylated and unphosphorylated state, respectively. These parameters are determined through the following coupled system:

2.2 $$\left[ {\begin{array}{*{20}l} {\dot{\alpha }_{A} } \hfill \\ {\dot{\alpha }_{B} } \hfill \\ {\dot{\alpha }_{C} } \hfill \\ {\dot{\alpha }_{D} } \hfill \\ \end{array} } \right] = \left[ {\begin{array}{*{20}l} {\quad - k_{1} } \hfill & {\quad k_{2} } \hfill & {\quad 0} \hfill & {\quad k_{7} } \hfill \\ {\quad k_{1} } \hfill & {\quad - \left( {k_{2} + k_{3} } \right)} \hfill & {\quad k_{4} } \hfill & {\quad 0} \hfill \\ {\quad 0} \hfill & {\quad k_{3} } \hfill & {\quad - \left( {k_{4} + k_{5} } \right)} \hfill & {\quad k_{6} } \hfill \\ {\quad 0} \hfill & {\quad 0} \hfill & {\quad k_{5} } \hfill & {\quad - \left( {k_{6} + k_{7} } \right)} \hfill \\ \end{array} } \right] \times \left[ {\begin{array}{*{20}l} {\alpha_{A} } \hfill \\ {\alpha_{B} } \hfill \\ {\alpha_{C} } \hfill \\ {\alpha_{D} } \hfill \\ \end{array} } \right]$$

where $\alpha$ represents different states of myosin and $k$ are transition rates between different myosin states. ${[{{\text{Ca}}}^{2+}]}_{i}$ will enter the system through ${k}_{1}$ and ${k}_{6}$:

2.3 $$k_{1} = k_{6} = \frac{{\left[ {{\text{Ca}}^{2 + } } \right]_{i}^{{nk_{1} }} }}{{\left[ {{\text{Ca}}^{2 + } } \right]_{i}^{{nk_{1} }} + \left[ {{\text{Ca}}_{{1/2{\text{MLCK}}}}^{2 + } } \right]_{i}^{{nk_{1} }} }}$$

where $[{{\text{Ca}}}_{1/2{\text{MLCK}}}^{2+}$]_*i*_ is the calcium ion concentration for half-activation of MLCK and $n{k}_{1}$ is the Hill coefficient.

Finally, the strain energy density function in the mechanical model, ${\Psi }_{{\text{uterus}}}$, is decoupled into the volumetric contribution ${\Psi }_{{\text{vol}}}$, the ground matrix isochoric contribution ${\overline{\Psi } }_{{\text{m}}}$, and the fiber isochoric contribution ${\overline{\Psi } }_{{\text{f}}}$:

2.4 $$\Psi_{{{\text{uterus}}}} = \Psi_{{{\text{vol}}}} + \overline{\Psi }_{{\text{m}}} + \overline{\Psi }_{{\text{f}}}$$

The volumetric contribution is defined by:

2.5 $$\Psi_{{{\text{vol}}}} = \frac{1}{{D_{1} }}\left( {\frac{{J^{2} - 1}}{2} - {\text{ln}}\left( J \right)} \right)$$

where ${D}_{1}$ is the incompressibility coefficient and $J$ is the volume ratio. The isochoric matrix contribution is given by the neo-Hookean model:

2.6 $${\overline{\Psi } }_{m}={C}_{10}({\overline{I} }_{1}-3)$$

where ${C}_{10}$ is a material parameter and ${\overline{I} }_{1}$ the first invariant of the isochoric right Cauchy–Green deformation tensor. Finally, the fiber isochoric contribution is calculated by:

2.7 $${\overline{\Psi } }_{f}={\zeta }_{1}\left({\overline{\Psi } }_{1}^{p}+{N}_{1}{\overline{\Psi } }_{1}^{a}\right)+(1-{\zeta }_{1})({\overline{\Psi } }_{2}^{p}+{N}_{2}{\overline{\Psi } }_{2}^{a})$$

where ${\zeta }_{1}$ represents the fraction of all fibers that belong to the first family, ${N}_{i}$ is a function that accounts for the overlap between actin and myosin filaments, and ${\overline{\Psi } }_{i}^{p}$ and ${\overline{\Psi } }_{i}^{a}$ represent the passive and active contribution of the *i*th fiber family, respectively. ${\overline{\Psi } }_{i}^{a}$ is defined by:

2.8 $${\overline{\Psi } }_{i}^{a}=\frac{1}{2}{\alpha }_{xb}{k}_{xb}{{\overline{\varepsilon }}_{e}}^{2}$$

where ${\alpha }_{xb}={\alpha }_{c}+{\alpha }_{D}$ establishes the connection between the chemical and mechanical model, ${k}_{xb}$ is a constant, and ${\overline{\varepsilon }}_{e}$ represents the elastic strain. The passive component ${\overline{\Psi } }_{i}^{p}$ is defined by:

2.9 $$\bar{\Psi }_{i}^{p} = \left\{ {\begin{array}{*{20}l} {\frac{{\mu _{{i1}} }}{{2\mu _{{i2}} }}\left\{ {{\text{exp}}\left[ {\mu _{{i2}} \left( {\bar{I}_{j} - 1} \right)^{2} } \right] - 1} \right\},} \hfill & {{\text{if}}\;\bar{I}_{j} \ge 1} \hfill \\ {0,} \hfill & {{\text{otherwise}}} \hfill \\ \end{array} } \right.$$

#### Membrane constitutive model

A modified version of the Buerzle–Mazza formulation (Buerzle and Mazza 2013) developed by Mauri and Ehret (Mauri et al. 2016a) was applied to characterize the mechanical response of the amnion (Mauri et al. 2016a). The strain energy function, ${\Psi }_{{\text{membrane}}}$, represents the strain energy per unit reference volume and accounts for the stretch of the single families of fibers (Buerzle and Mazza 2013; Mauri et al. 2016a):

2.10 $${\Psi }_{{\text{membrane}}}=\frac{{\mu }_{0}}{2q}[{e}^{qg}-1]$$

where ${\mu }_{0}$ is a material constant having the units of stress and $q$ is a dimensionless constant that controls the nonlinearity of the moduli for the different stress responses in the composite structure (Rubin and Bodner 2002).

The function $g$ is given by the sum of the compressible neo-Hookean material ${g}_{2}$ and the fiber strain energy ${g}_{3}$ (Buerzle and Mazza 2013; Mauri et al. 2016a):

2.11 $$g={g}_{2}\left({I}_{1}, J\right)+{g}_{3}\left({\lambda }_{i}\right)$$

2.12 $${g}_{2}={m}_{2}\left({I}_{1}-3\right)+\frac{{m}_{2}}{{m}_{5}}({J}^{-2{m}_{5}}-1)$$

2.13 $$g_{3} = \frac{{m_{3} }}{{m_{4} }}\frac{1}{N}\mathop \sum \limits_{i = 1}^{N} \left\langle {\lambda_{i} - 1} \right\rangle^{{2m_{4} }}$$

where $\langle \rangle$ represents the Macauley brackets, which indicates that the fibers do not bear compression loads (Buerzle and Mazza 2013), and ${m}_{i}$ are model parameters. The parameter $N$ represents the number of representative families and must be even and greater than zero. The first invariant of the right Cauchy–Green deformation tensor is represented by ${I}_{1}$, while $J$ is the volume ratio, and ${\lambda }_{i}$ is the fiber stretch (Buerzle and Mazza 2013; Mauri et al. 2016a):

2.14 $${I}_{1}=tr({{\varvec{F}}}^{T}{\varvec{F}})$$

2.15 $$J={\text{det}}{\varvec{F}}$$

2.16 $${\lambda }_{i}=\left|{\varvec{F}}{{\varvec{M}}}_{i}\right|, i=\mathrm{1,2},\dots ,N$$

The deformation gradient represented by ${\varvec{F}}$ and ${\varvec{M}}$ represents the fiber directions in the reference configuration and it is defined as (Mauri et al. 2016a):

2.17 $${{\varvec{M}}}_{j}={\text{cos}}{\beta }_{j}{\text{sin}}{\theta }_{j}{{\varvec{e}}}_{1}+{\text{sin}}{\beta }_{j}{\text{sin}}{\theta }_{j}{{\varvec{e}}}_{2}+{\text{cos}}{\theta }_{j}{\varvec{N}}$$

2.18 $${{\varvec{M}}}_{j+N/2}={\text{cos}}{\beta }_{j}{\text{sin}}{\theta }_{j}{{\varvec{e}}}_{1}+{\text{sin}}{\beta }_{j}{\text{sin}}{\theta }_{j}{{\varvec{e}}}_{2}-{\text{cos}}{\theta }_{j}{\varvec{N}}$$

2.19 $${\beta }_{j}=\frac{2\pi }{N}(j-\frac{3}{2})$$

2.20 $${\theta }_{j}=\frac{\pi }{2}-\phi$$

where $\phi$ represents a slight off-plane inclination and $j=\mathrm{1,2},\dots ,\frac{N}{2}$. The Cauchy stress tensor ${\varvec{\upsigma}}$ is obtained from the derivatives of the strain energy density function with respect to the deformation tensor (Buerzle and Mazza 2013).

Eight parameters must be defined to fully characterize the material response: ${\mu }_{0}$, $q$, ${m}_{2}$, ${m}_{3}$, ${m}_{4}$, ${m}_{5}$, $N$, and $\phi$.

### Parametrized numerical model of the gravid uterine body and cervix

**Fig. 2:**
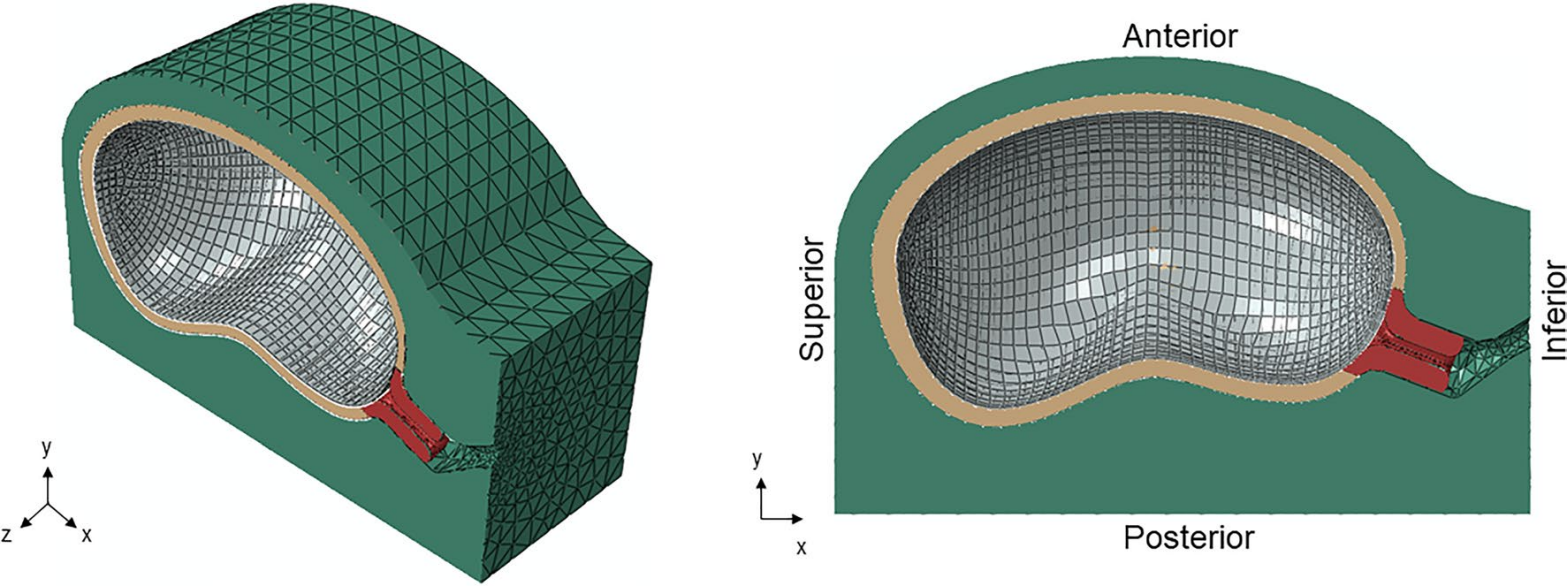
Parametrized finite element model of the gravid uterine body and cervix. The model comprises four structures: body of the uterus (brown), cervix (red), multilayer fetal membrane (gray), and abdomen (green). The anatomical directions (anterior, posterior, superior, and inferior) are also represented

A complete parametrized numerical model of the gravid uterine body and cervix (Fig. 2) was developed to accomplish the objectives of this work, following the instructions of Louwagie et al. (Louwagie et al. 2021). Since the geometric model is parametrized, it is possible to generate patient-specific geometries by changing the required dimensions. The model was developed for a mid-third-trimester gravid uterine body and cervix, using the average dimensions from different patients retrieved from Louwagie et al. (Louwagie et al. 2021). The model comprises four structures: the body of the uterus, cervix, multilayer fetal membrane (amnion, chorion, and part of the decidua), and abdomen. SubSects. 2.2.1, 2.2.2, 2.2.3, 2.2.4 present a more detailed description of each structure and the respective finite element characterization. In subSect. 2.2.5, the complete model’s loads, contact interactions, and boundary conditions are described.

#### Body of the uterus

**Fig. 3:**
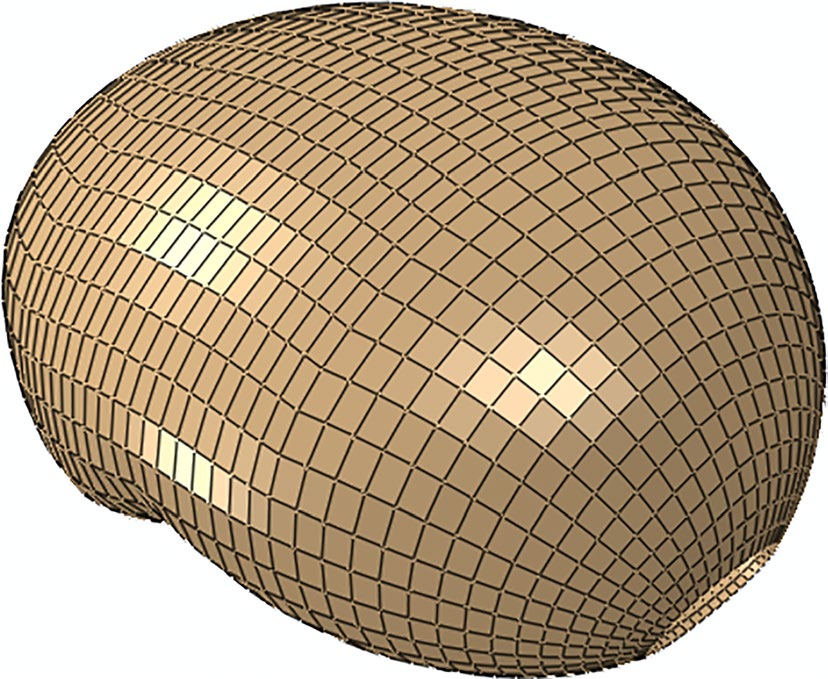
Finite element model of the uterine cavity, meshed with C3D8H finite elements

The finite element model of the uterine body (Fig. 3) contains 6333 C3D8H hybrid linear brick finite elements and 8536 nodes.

In the longitudinal and circumferential directions, two families of uterine collagen fibers were considered. The direction of each fiber was found through the finite element software ABAQUS®, applying the methodology described in previous works (Fidalgo et al. 2022).

The electro-chemo-mechanical constitutive model characterized the uterine body, able to reproduce muscle contractions, as described in Table 1. The electrical component was calibrated using transmembrane potential and calcium concentration curves (Sharifimajd and Stålhand 2014). The chemical parameters were calibrated using the myosin states and stress evolutions (Sharifimajd et al. 2016). The mechanical parameters were calibrated using an intrauterine pressure curve measured in a woman at term (38 weeks) in active labor (Sharifimajd et al. 2016).

**Table 1:** Electrical, chemical, and mechanical properties of the uterine body, using the electro-chemo-mechanical constitutive model

	Variable	Description	Value	Units
Electrical parameters (Rihana et al. 2009; Yochum et al. 2016)	*I*_stm_	Stimulation current	0.17	μA/cm^2^
	*C*_m_	Membrane capacitance	1	μF
	*f*_c_	Calcium influx probability	0.4	–
	*Α*	Current conservation factor	4 × 10^−5^	mol cm^2^/μC
	*K*_Ca_	Calcium extraction factor	0.01	mseg^−1^
	*J*_back_	Background calcium current	0.023	μA/cm^2^
	*G*_Ca_	Voltage-operated calcium channel conductance	0.022	mS/cm^2^
	*V*_Ca_	Half-point of the voltage operated channel activation sigmoid	− 20	mV
	*R*_Ca_	Maximum slope of the voltage operated channel activation	5.97	mV
	*G*_k_	Potassium channel conductance	0.064	mS/cm^2^
	*E*_K_	Potassium Nernst potential	− 83	mV
	*K*	Half-point potassium concentration	0.01	μmol
	*G*_KCa_	Potassium/calcium channel conductance	0.08	mS/cm^2^
	*G*_L_	Leak channel conductance	0.0055	mS/cm^2^
	*E*_L_	Leak Nernst potential	− 20	mV
	[Ca^2+^]_e_	Extracellular calcium concentration	2	μmol
	*R*	Gas constant	8.134	JK^−1^mol^−1^
	*T*	Temperature	295	K
	*F*	Faraday constant	96.487	kCmol^−1^
Chemical parameters (Sharifimajd et al. 2016)	*k*_2_	Dephosphorylation of myosin rate	1.2387	seg^−1^
	*k*_3_	Attachment and detachment of the fast cycling cross-bridge rate	0.1419	seg^−1^
	*k*_7_	Latch bridge detachment rate	0.0378	seg^−1^
	$[{{\text{Ca}}}_{1/2{\text{MLCK}}}^{2+}$]_i_	Calcium ion concentration for half-activation of MLCK	2.5698 × 10^–4^	mM
	*n*_*k1*_	Hill coefficient	8.7613	–
Mechanical parameters (Sharifimajd et al. 2016)	*C*_10_	Stiffness coefficient	0.030	MPa
	*D*_10_	Volumetric coefficient	1E-5	MPa^−1^
	ζ_1_	Fraction of fibers of the first family	1	–
	μ_i1_ (i = 1,2)	Fiber’s Stiffness	0.006	MPa
	*μ*_*i*2_ (*i* = 1,2)	Fiber’s nonlinear behavior	1	–
	${\overline{\varepsilon }}_{{\text{opt}}}$	Active strain for maximum force	0.4950	–
	$\gamma$	Constant	1	–
	${\nu }_{{\text{C}}}$	Peripheral clutch velocity	0.11	seg^−1^
	$\eta$	Constant	18	MPa s
	${k}_{xb}$	Constant	6	MPa
	${k}_{{\text{C}}}$	Constant	3.5	MPa s

#### Cervix

**Fig. 4:**
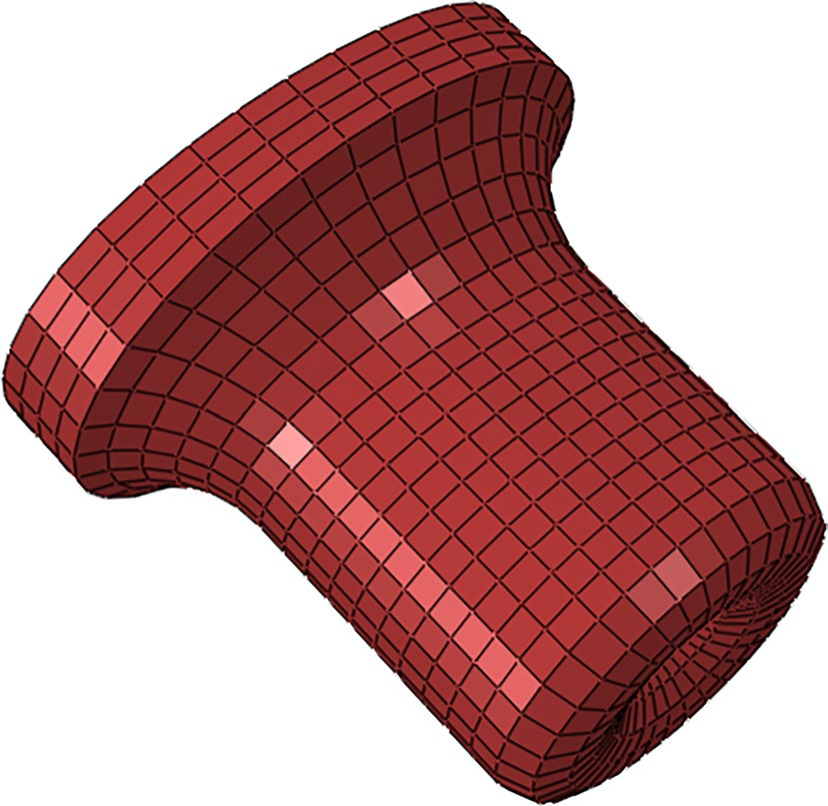
Finite element model of the cervix, meshed with C3D8H finite elements

The cervix was modeled with C3D8H hybrid linear brick finite elements, using 3910 elements and 4794 nodes, as illustrated in Fig. 4.

Collagen fibers in the longitudinal direction (from the lower to the upper region of the cervix) and circumferential direction (around the cervical canal) were introduced into the cervical model, following the methodology described for the uterus.

The electro-chemo-mechanical model was also applied to describe the material response of the cervix. The parameters were maintained according to Table 1, except for ${C}_{10}$ and ${D}_{10}$, which were adjusted to the tensile properties of non-pregnant human cervical tissue (Fernandez et al. 2016):

$$C_{10} = 0.186 \;{\text{MPa}}$$

$$D_{10} = 2.48 \;{\text{MPa}}^{ - 1}$$

Active cervical contractions were produced by activating the electrical and chemical components. To simulate a passive cervix, these sub-models were inactivated.

#### Fetal membrane

**Fig. 5:**
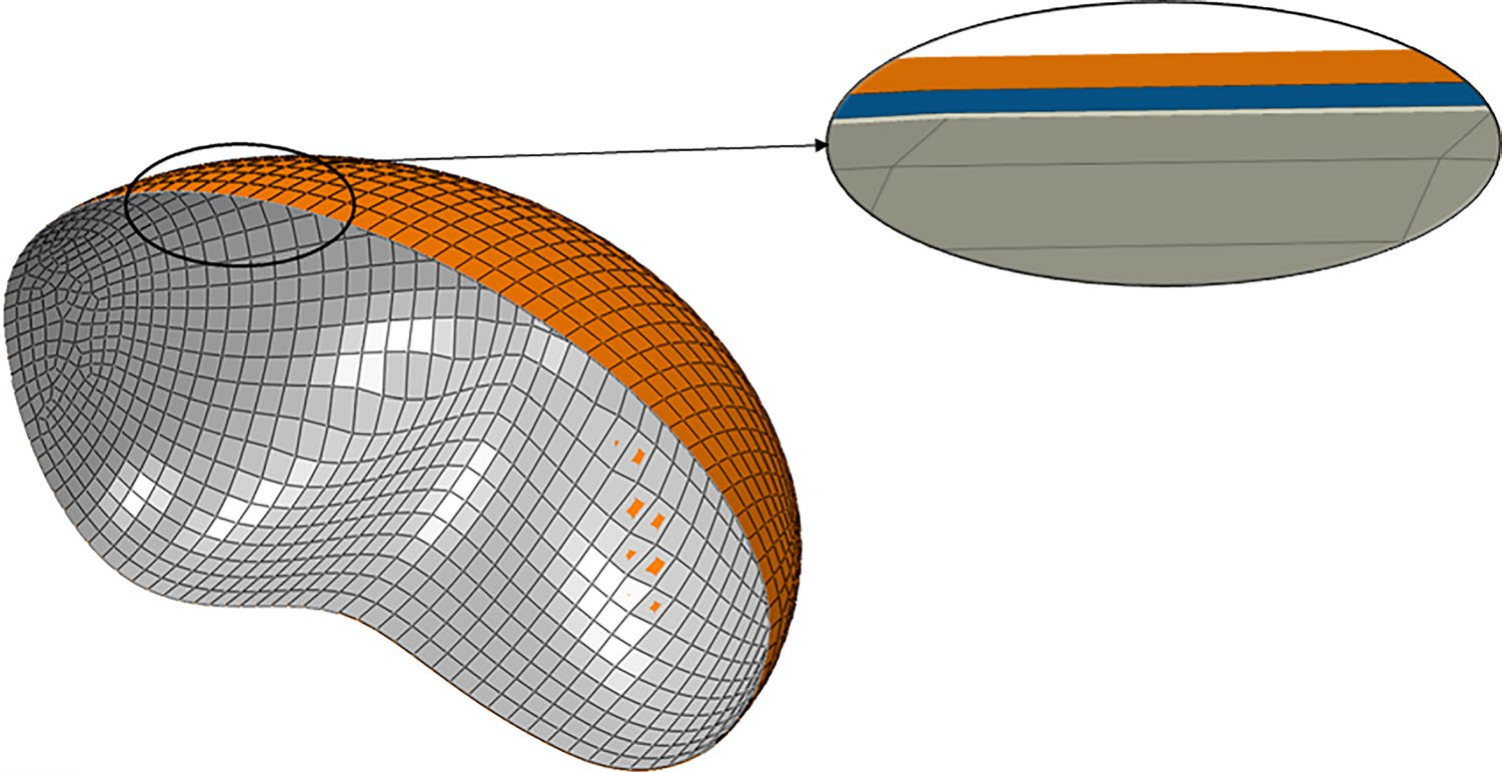
Finite element model of the multilayer fetal membrane, meshed with C3D8(H) finite elements: amnion (gray), chorion (blue), and decidua (orange)

In this work, a multilayer fetal membrane model was developed, comprising the amnion, chorion, and part of the decidua. The fetal membrane is illustrated in Fig. 5, and it was modeled with linear brick finite elements: The amnion comprised 2874 C3D8H and 8628 nodes, the chorion contained 2517 C3D8 elements and 7557 nodes, and the decidua was modeled with 2609 C3D8 elements and 7833 nodes.

The amnion, the mechanically dominant layer of the fetal membrane, was characterized by the modified version of the Buerzle–Mazza formulation (Buerzle and Mazza 2013). In Table 2, the mechanical parameters of the amnion layer are presented.

**Table 2:** Mechanical properties of the amnion, using the modified version of the Buerzle–Mazza constitutive model (Fidalgo et al. 2024; Mauri et al. 2016a)

	${\mu }_{0}$ (MPa)	$q$	${m}_{2}$	${m}_{3}$	${m}_{4}$	${m}_{5}$	$N$	$\phi$
Amnion	2.4	2.96	0.00228	41.12	1.27	0.463	32	0

Since the chorion and decidua layers are less mechanically dominant than the amnion layer, both layers were characterized as linear elastic materials (Table 3). It was hypothesized that the chorion and the decidua share similar mechanical properties. It is known that the decidua shares a close anatomical relationship with the chorion, suggesting that decidual changes may be associated with chorioamnion membrane alterations (Malak and Bell 1994). The development of the decidual layer and the chorion is synchronized from the earliest phase of implantation, and the smooth chorion becomes fused with the decidua (Ogura et al. 2017).

**Table 3:** Mechanical properties of the chorion and decidua, using the linear elastic constitutive model (Ophir et al. 2002; Oxlund et al. 1990; Roohbakhshan et al. 2016; Verbruggen et al. 2017)

	$E$ (MPa)	$\nu$ [–]
Chorion	1	0.41
Decidua	1	0.49

#### Abdomen

**Fig. 6:**
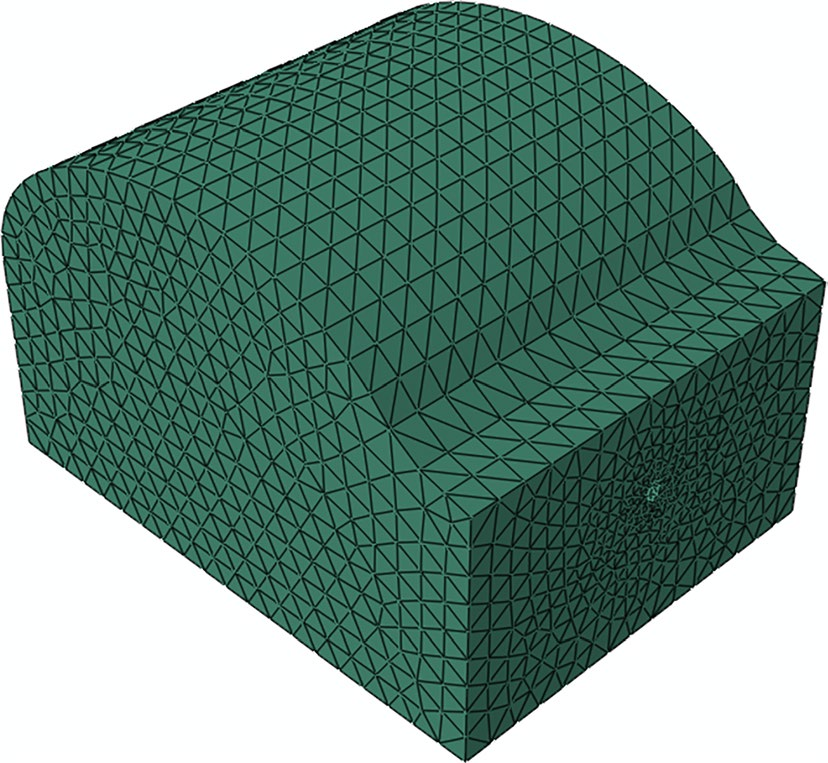
Finite element model of the abdomen, meshed with C3D4H finite elements

The finite element model of the abdomen is illustrated in Fig. 6. This structure was meshed with 27,836 C3D4H hybrid tetrahedral finite elements and 5887 nodes.

The neo-Hookean constitutive model was used to characterize the abdomen, and the respective mechanical parameters were set to the values in Table 4.

**Table 4:** Mechanical properties of the abdomen, using the neo-Hookean constitutive model (Westervelt et al. 2017)

	${C}_{10}$ (MPa)	${D}_{10}$ (MPa^−1^)
Abdomen	0.005	1E-5

#### Loads, contact interactions, and boundary conditions

**Fig. 7:**
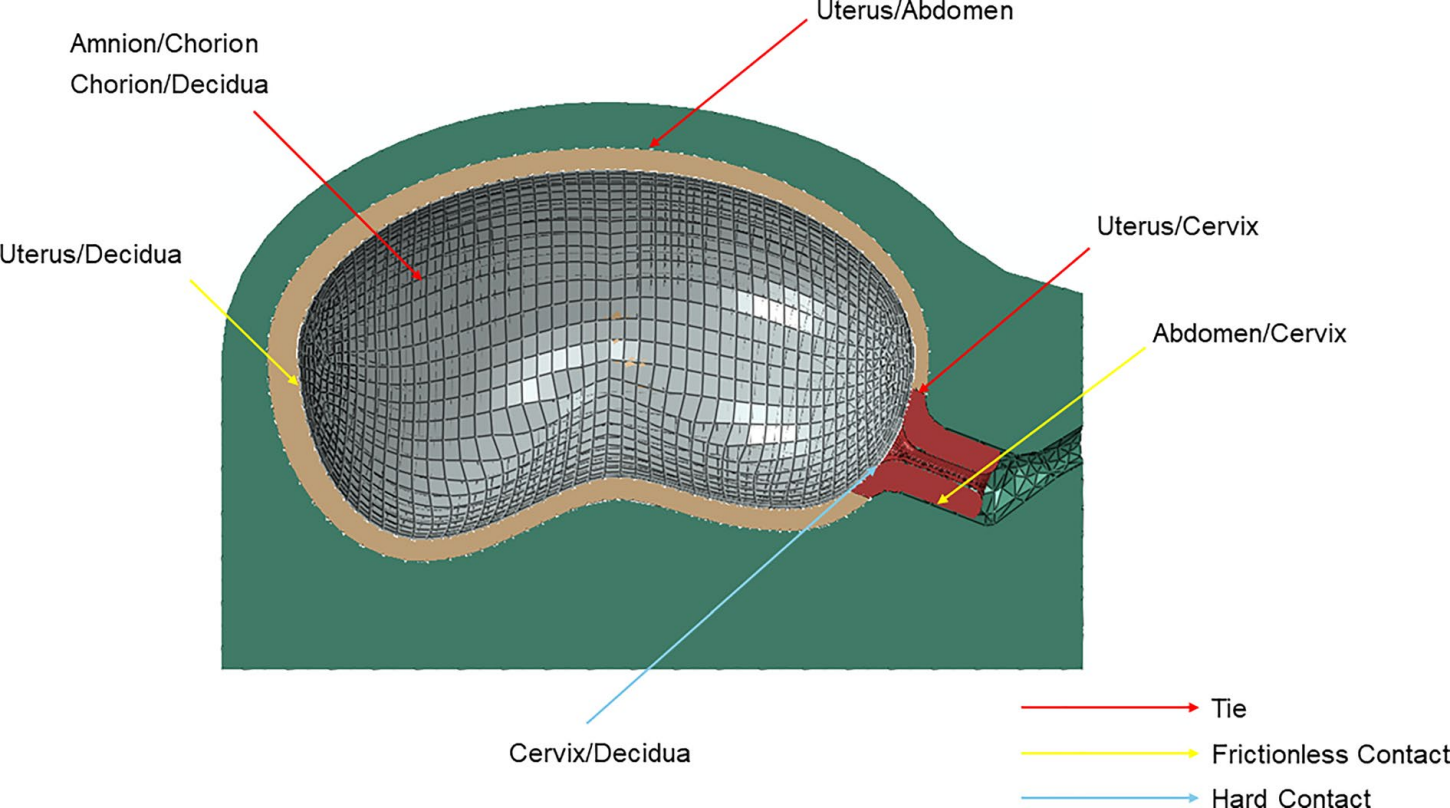
Contact interaction defined in the biomechanical model

Due to the complexity of the finite element model of the gravid uterine body and cervix (Fig. 2), the contact interactions were carefully analyzed and defined according to previous works. Three types of contact interactions were considered (Fig. 7):

- *Tie contact* (body of the uterus/abdomen; amnion/chorion; chorion/decidua; body of the uterus/cervix);
- *Frictionless contact* (cervix/abdomen; body of the uterus/decidua);
- *Hard contact* (decidua/cervix).

Regarding the load conditions applied in the numerical model, a fluid cavity representing the amniotic fluid was created inside the uterine body, where a ramping intrauterine pressure was defined. The fluid density was set to 1.025 kg/m^3^, and the fluid bulk modulus to 2200 MPa, whose values are from blood plasma (Orczyk-Pawilowicz et al. 2016). The intrauterine pressure was set to the labor contraction value of 0.00867 MPa (Grimm 2021).

**Fig. 8:**
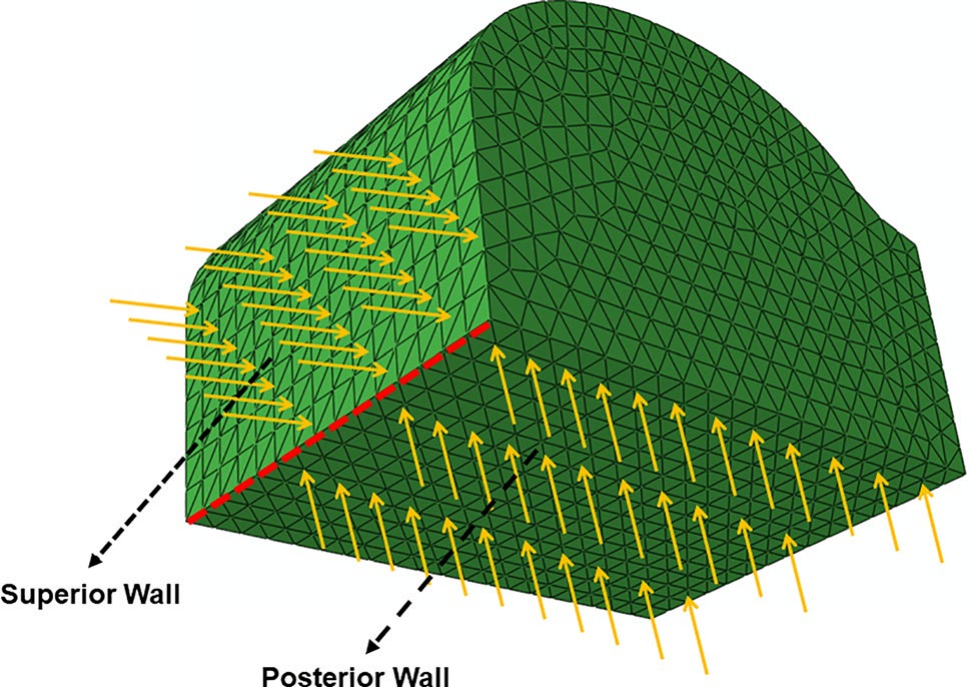
Boundary conditions applied to the posterior and superior surfaces of the abdomen; the yellow arrows represent the restricted directions and the red line represent a restriction in all directions

Finally, three boundary conditions were established according to Fig. 8:

- The nodes located in the posterior wall of the abdomen were restricted in the direction perpendicular to the respective surface;
- The nodes located in the superior wall of the abdomen were restricted in the direction perpendicular to the respective surface;
- The nodes in common between the posterior and superior walls of the abdomen were restricted in all directions.

### Numerical simulations

The numerical model of the gravid uterine body and cervix was used to study the interaction between the cervix, fetal membrane, and uterine contractions. The following aspects were remained equal across all numerical simulations:

- The body of the uterus was always contracting;
- All simulations had a duration of a normal contraction time (80 s) (Buttin et al. 2013).

Four physiological factors associated with the gestation period were investigated to identify their impact on the fast cervical shape change before labor (dilation and shortening) and the fetal membrane rupture: (i) initial cervical shape, (ii) cervical stiffness, (iii) cervical contraction, and (iv) intrauterine pressure. Table 5 summarizes all simulations and an explanation is presented in the following paragraphs.

**Table 5:** Simulations performed to investigate cervical shape, cervical stiffness, cervical contraction, and IUP

Factors	Cervical shape			Cervical stiffness (MPa)			Cervical contraction		IUP (MPa)		
	Large	Mid	Small	0.186	0.070	0.020	Yes	No	0.015	0.00867	0.005
Cervical shape and cervical stiffness	X			X				X		X	
	X				X			X		X	
	X					X		X		X	
		X		X				X		X	
		X			X			X		X	
		X				X		X		X	
			X	X				X		X	
			X		X			X		X	
			X			X		X		X	
Cervical contraction	X					X	X			X	
		X				X	X			X	
			X			X	X			X	
IUP	X					X		X	X		
	X					X		X		X	
	X					X		X			X

**Fig. 9:**
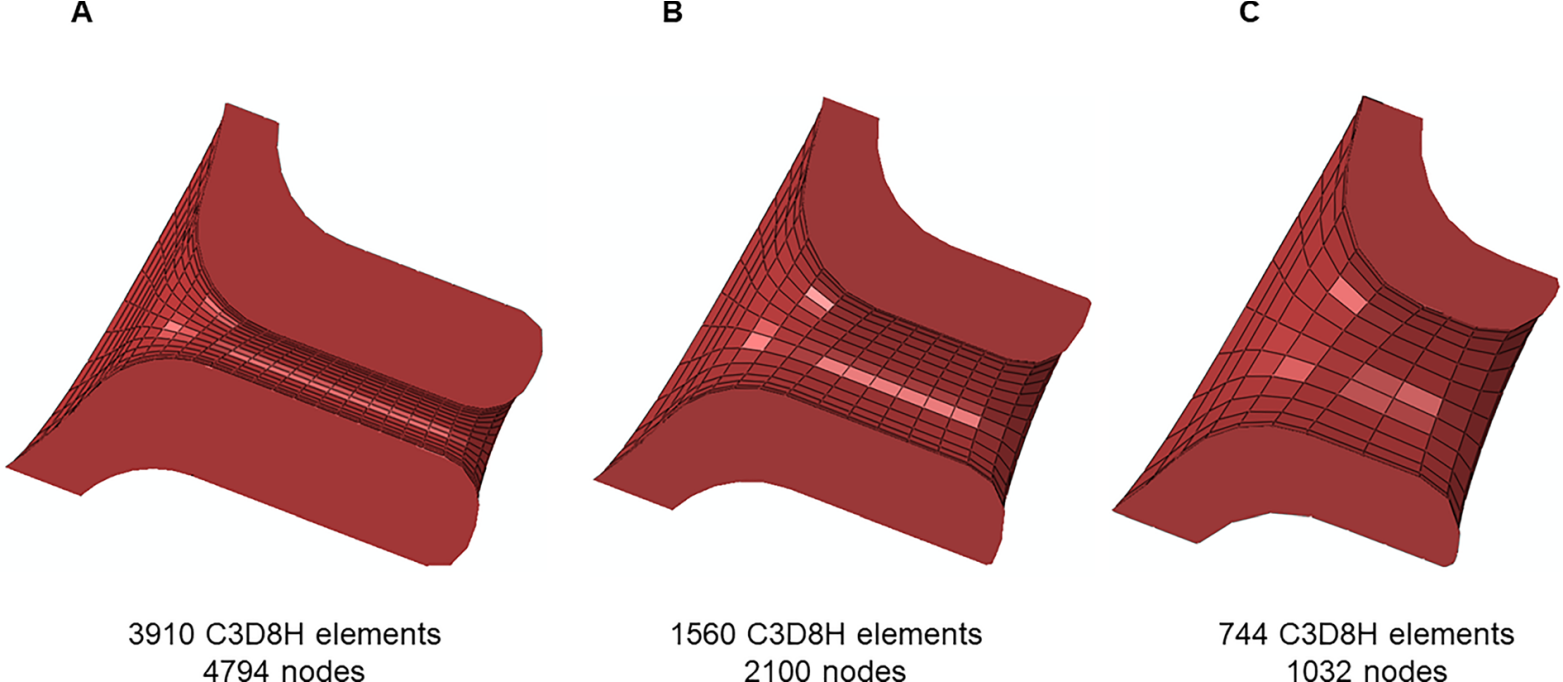
Different initial cervical shapes were analyzed in the numerical simulations: **A** “Large” shape, **B** “Mid” shape, and **C** “Small” shape

Regarding the initial cervical shape (i), three scenarios were considered (Louwagie et al. 2021):

- “Large” Shape—large cervical length (35 mm) and small cervical dilation (inner diameter of the cervical canal set to 5 mm) (Fig. 9A);
- “Mid” Shape—mid-cervical length (28 mm) and mid-dilation (inner diameter of the cervical canal set to 10 mm) (Fig. 9B);
- “Small” Shape—small cervical length (20 mm) and large cervical dilation (inner diameter of the cervical canal set to 15 mm) (Fig. 9C);

Therefore, the cervical shape got shorter and more dilated from Fig. 9A–C.

For each shape illustrated in Fig. 9, the stiffness parameter ${C}_{10}$ from the cervix (ii) was set to three different values: 0.186 MPa (subSect. 2.2.2), 0.070 MPa (reduction of 62%), and 0.020 MPa (reduction of 89%). This accounted for nine numerical simulations: 3 cervical stiffness values per 3 cervical shapes. The cervix did not contract during these nine simulations, and the intrauterine pressure was maintained to be equal to 000867 MPa.

Then, the electrical and chemical parts of the electro-chemo-mechanical model were activated for the cervix (iii) to evaluate the impact of cervical contractions on cervical shortening and dilation. Three more numerical simulations were performed for each cervical shape, with ${C}_{10}$ set to 0.02 MPa. The intrauterine pressure was maintained equal to 0.00867 MPa.

The intrauterine pressure (IUP) effect on cervical shape change and fetal membrane rupture (iv) was also contemplated in this work. Two more simulations were developed, setting the intrauterine pressure to 0.005 MPa and 0.015 MPa. These IUPs were tested in the cervix from “Large” Shape, with ${C}_{10}$ equal to 0.02 MPa.

**Fig. 10:**
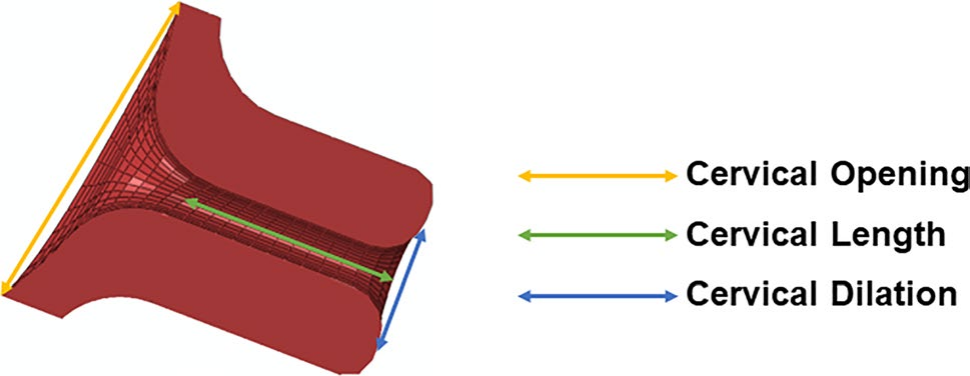
Cervical measurements analyzed in the results section

To analyze the cervical shape change for each simulation, three cervical measurements were defined as illustrated in Fig. 10:

- Variation of cervical opening (CO);
- Variation of cervical length (CL);
- Variation of cervical dilation (CD).

These measurements represent the difference between the final and initial cervical configurations (*e.g.,* if the initial cervical length equals 20.00 mm and the final length equals 19.80 mm CL = 19.80–20.00 =  − 0.20 mm).

**Fig. 11:**
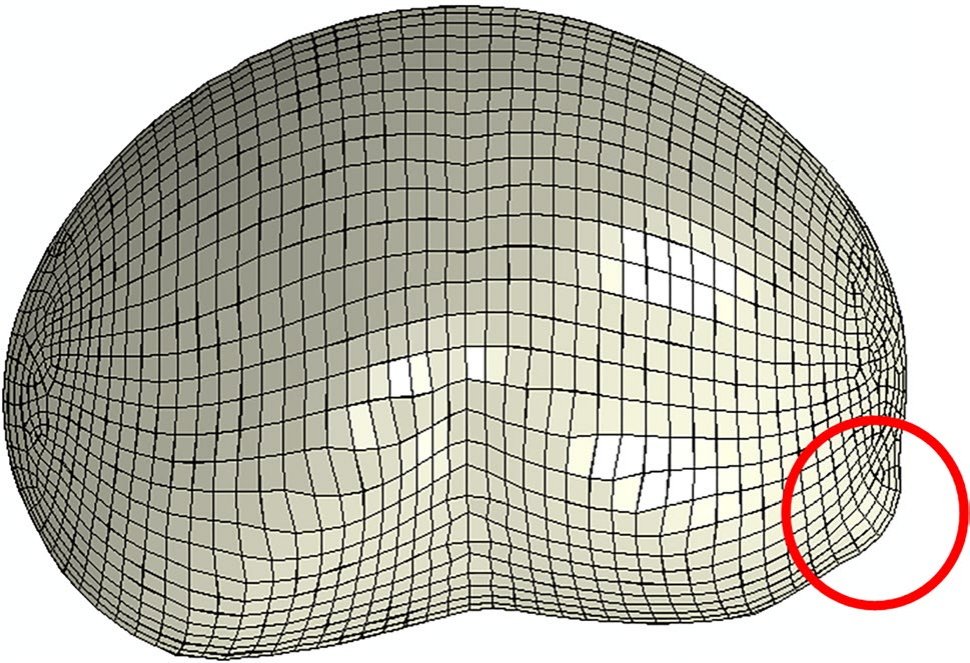
The amnion's protrusion is called the "weak zone" (highlighted in red), where the fetal membrane rupture initiates

Finally, the fetal membrane rupture was analyzed by retrieving the maximum principal stress in the amnion layer’s protrusion, called the “weak zone” (Fig. 11), and compared with the respective biaxial strength in the discussion section. The protrusion occurs in loaded configurations when the amnion is pushed against the cervical opening. The maximum principal stresses were not retrieved for the chorion and decidua since they do not represent the mechanically dominant layers of the fetal membrane.

### Convergence study

**Fig. 12:**
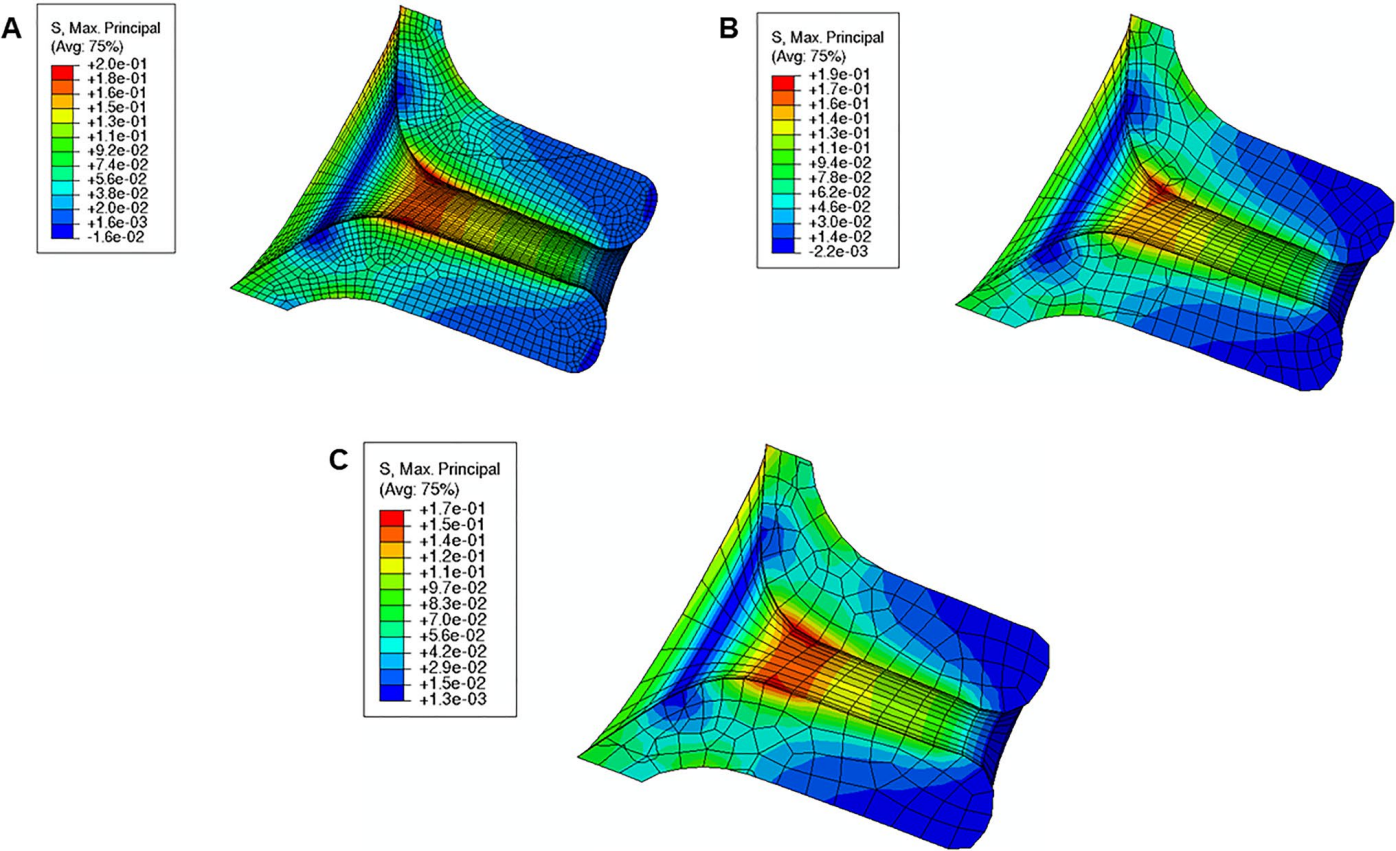
Convergence study on the cervix: **A** very refined mesh, **B** refined mesh, and **C** coarse mesh

The cervix assumed different geometries and stiffnesses throughout this investigation. A convergence study was therefore performed, resorting to a simplistic model of the cervix. A pressure of 0.1 MPa was applied to the cervical canal and displacements were restricted in the surface contacting the uterine body in the gravid model. Three simulations were performed using three different finite element meshes (Fig. 12): (A) very refined mesh, (B) refined mesh, and (C) coarse mesh.

The maximum principal stresses obtained with a refined mesh (Fig. 12B) are similar to the stresses obtained with a very refined mesh, while a coarse mesh led to a larger discrepancy. Since the full-gravid uterine model is very complex, the cervix was meshed as illustrated in Fig. 12B, to reduce computational costs associated with a very refined mesh without compromising the results. The average finite element size was maintained as close as possible between different cervical geometries.

## Results

Numerical simulation results are illustrated and described in this section based on maximum principal stress distribution in the uterus, fetal membrane, and cervix (especially the internal os region), and cervical shape change.

### Uterine body and fetal membrane

**Fig. 13:**
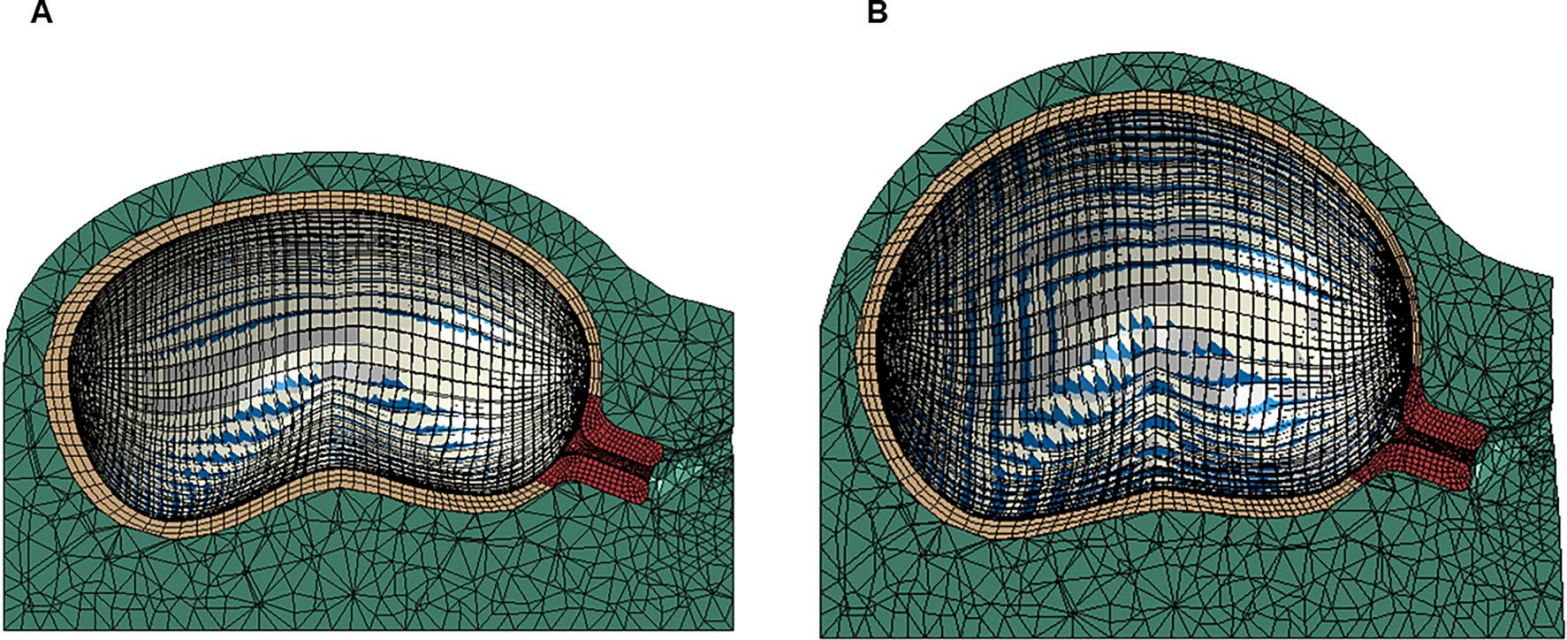
Interaction between the cervix, fetal membrane, and body of the uterus: **A** reference configuration; **B** deformed configuration

The reference and deformed configuration of the entire model is illustrated in Fig. 13 for one of the simulations.

**Fig. 14:**
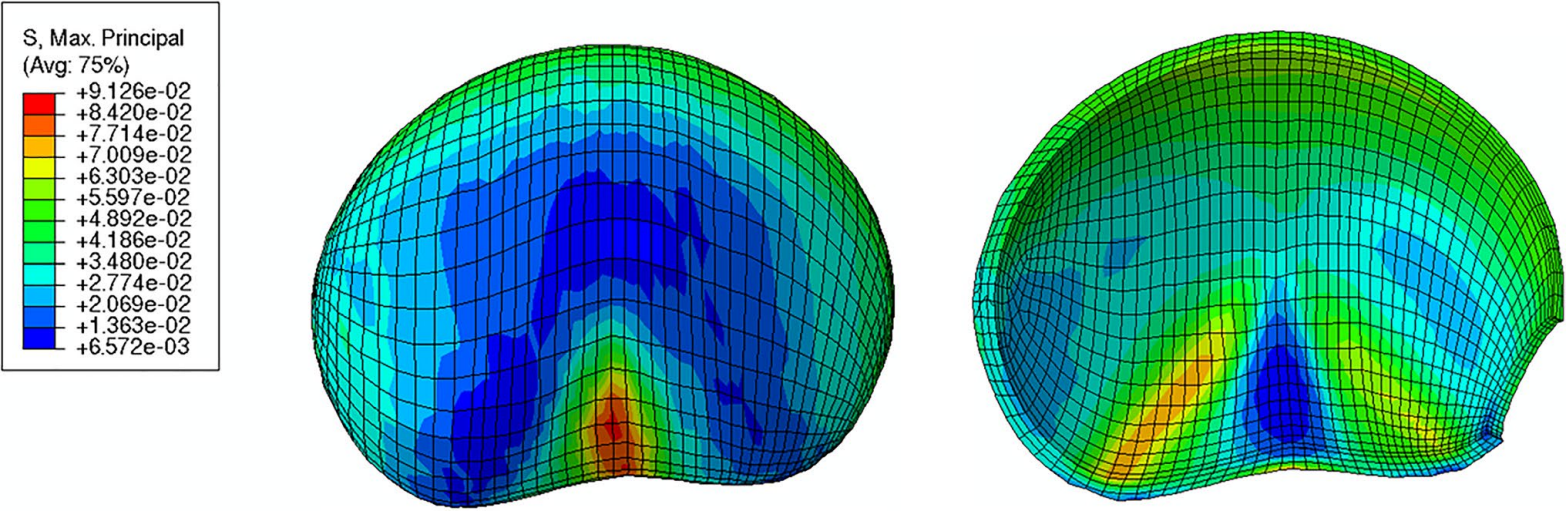
Maximum principal stress in the uterine muscle (units: MPa)

**Fig. 15:**
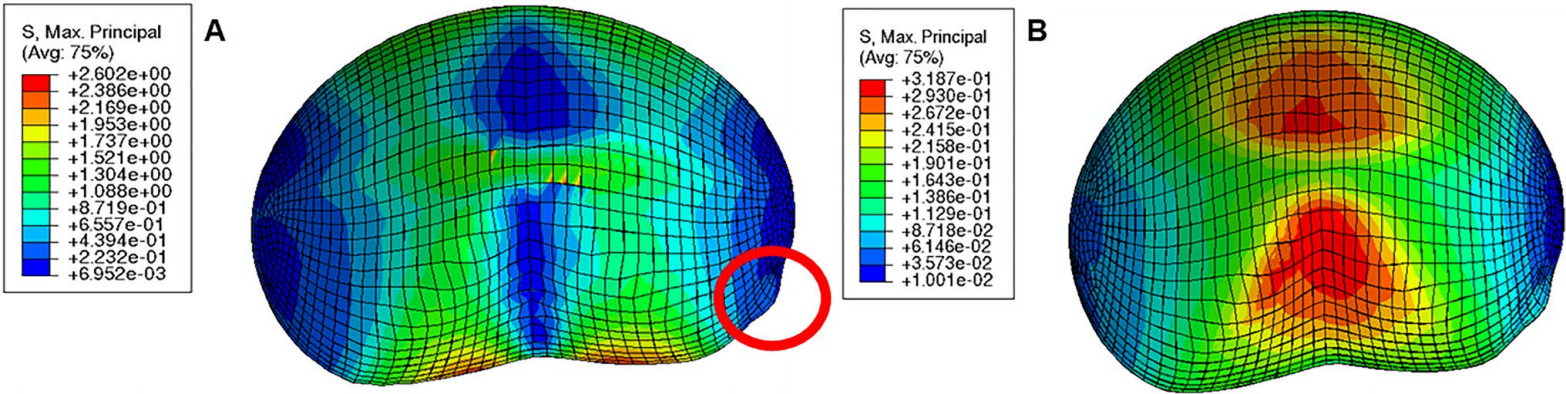
Maximum principal stress in the amnion **A** and chorion **B** layers (units: MPa). The red circle represents the "weak zone" from the fetal membrane in the amnion layer

The maximum principal stress values and distributions in the body of the uterus, amnion, and chorion are represented in Figs. 14 and 15. Those values and distributions are similar across all simulations, except for those where the IUP is modified.

According to Fig. 14, the maximum principal stress in the uterine muscle is verified at the center of the posterior region and corresponds to a value of 0.091 MPa. The uterine contractions cause the uterine body to contract longitudinally and expand circumferentially.

Focusing on the main layers of the fetal membrane (amnion and chorion), illustrated in Fig. 15, the amnion is submitted to much larger maximum principal stresses than the chorion. The maximum stress value in the amnion is 2.602 MPa, while in the chorion is 0.319 MPa.

### Initial cervical shape and cervical stiffness

**Fig. 16:**
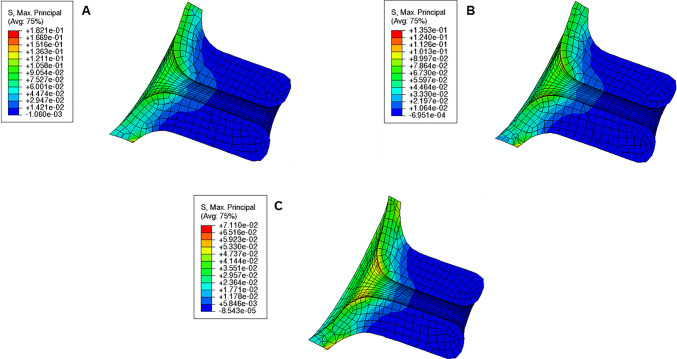
Maximum principal stress distributions for the cervical shape described in “Large” shape (units: MPa); **A**: stiff cervix (${C}_{10}=0.186$ MPa); **B**: mid-cervical stiffness (${C}_{10}=0.07$ MPa), **C**: soft cervix (${C}_{10}=0.02$ MPa)

**Fig. 17:**
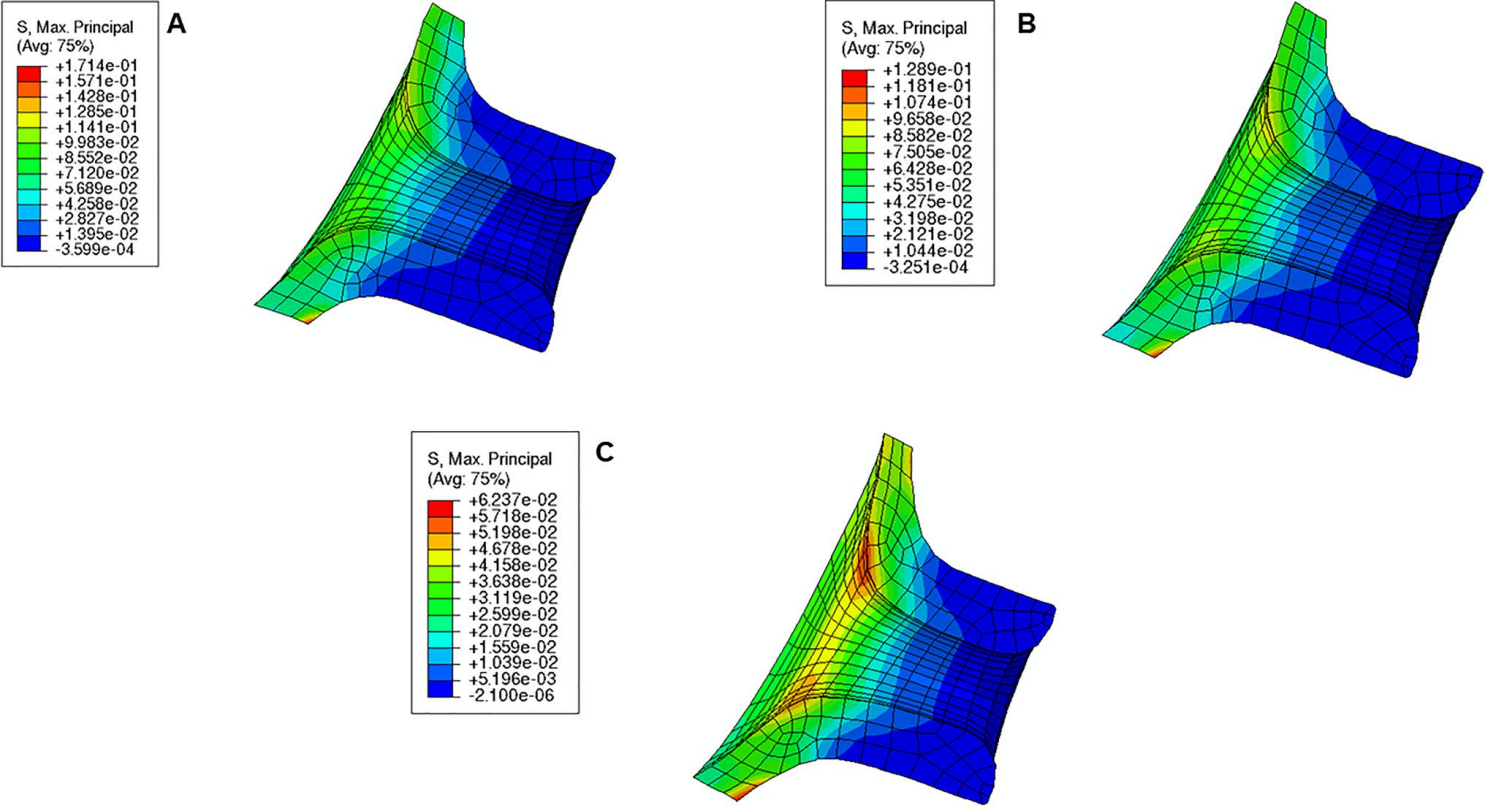
Maximum principal stress distributions for the cervical shape described in “Mid” Shape [units: MPa]; A: stiff cervix (${C}_{10}=0.186$ MPa); B: mid-cervical stiffness (${C}_{10}=0.07$ MPa), C: soft cervix (${C}_{10}=0.02$ MPa)

**Fig. 18:**
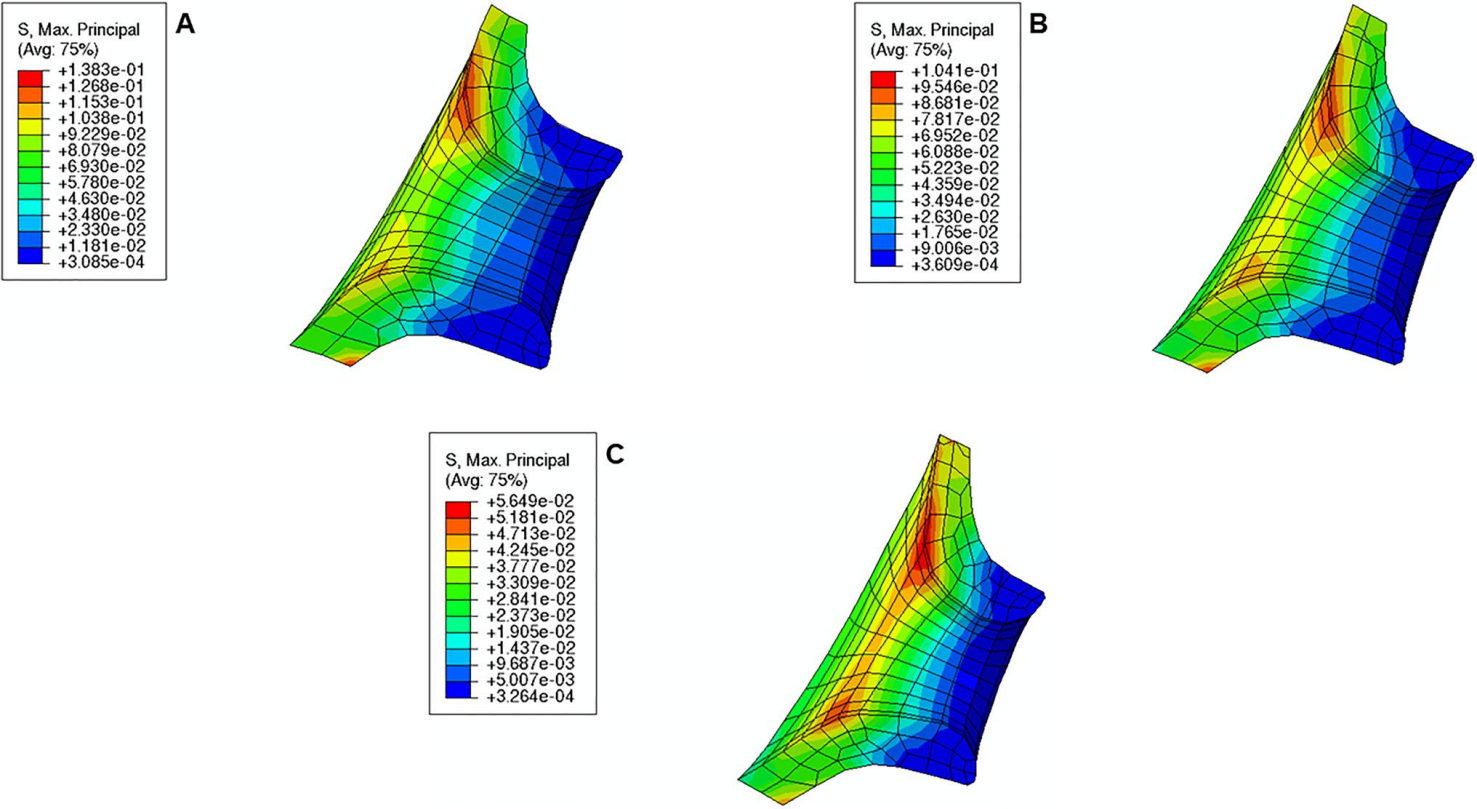
Maximum principal stress distributions for the cervical shape described in “Small” Shape [units: MPa]; A: stiff cervix (${C}_{10}=0.186$ MPa); B: mid-cervical stiffness (${C}_{10}=0.07$ MPa), C: soft cervix (${C}_{10}=0.02$ MPa)

The maximum principal stress in the cervix tends to decrease as the reference configuration gets shorter and more dilated, indicating that stress values are influenced by initial cervical shape (Figs. 16, 17, and 18; cervices with equal stiffness values are identified by the same letter A, B, or C).

The stress values decrease for the same initial cervical shape as the cervix becomes softer (lower ${C}_{10}$ values). Moreover, stress concentrations are found for all cases at the internal *os* region, considered the initial site of cervical opening.

The largest maximum principal stress value is equal to 0.182 MPa, verified in Fig. 16A, which corresponds to a long, more closed, and stiff cervix. On the other hand, the smallest maximum principal stress value is 0.056 MPa, verified in Fig. 18C, where a short, dilated, and soft cervix is illustrated. This corresponds to a decrease of 69.2% in terms of maximum principal stress, highlighting how impactful cervical shape and stiffness are for cervical stresses.

**Fig. 19:**
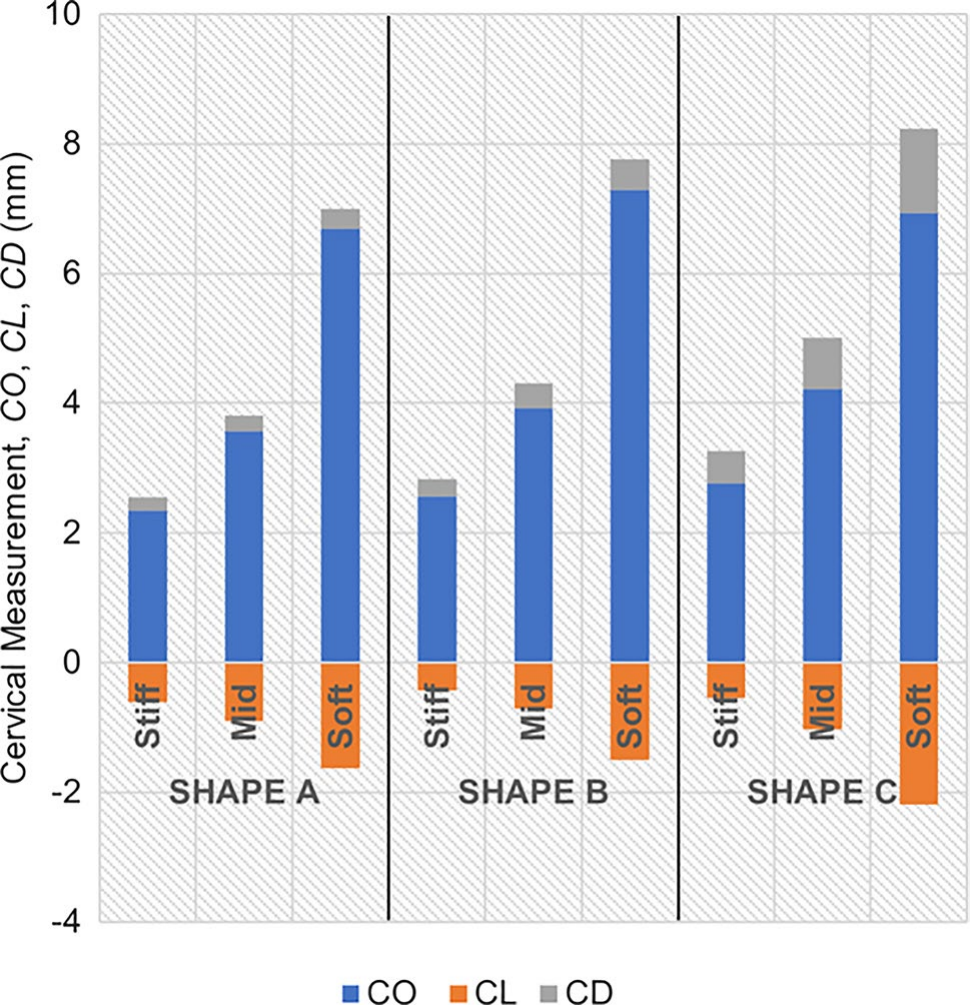
Variations of cervical opening (CO), cervical length (CL), and cervical dilation (CD) for different cervical shapes and stiffnesses. “Large” shape, “Mid” shape, and “Small” shape represent different cervical shapes as described in Sect. 2.3, while “Stiff,” “Mid,” and “Soft” represent different cervical stiffness values

From the analysis of Fig. 19, the following observations are valid:

- For each cervical shape, the absolute values of CO, CL, and CD increase as cervical stiffness decreases;
- CO and CD are always positive, which means that the respective measurements increase relative to the initial cervical configuration; the opposite observation is obtained for CL;
- the absolute values of CL and CD are maximum for the “Soft” cervix from “Small” Shape; CO is maximum for the “Soft” cervix from “Mid” Shape;
- CO and CD are minimal for the “Stiff” cervix from “Large” Shape; the absolute value of CL is minimal for the “Stiff” cervix from “Mid” Shape;
- comparing the “Stiff” with the “Soft” cervix, the largest numerical difference for CO is verified in “Mid” Shape, and for CL and CD in “Small” Shape.

Finally, in terms of maximum principal stress at the “weak zone” of the amnion layer, the following values are retrieved:

- 0.3 MPa for “Large” Shape, independent of the cervical stiffness;
- 0.5 MPa for “Mid” Shape, independent of the cervical stiffness;
- 0.7 MPa for the “Stiff” and “Mid” cervices from “Small” Shape; 0.8 MPa for the “Soft” cervix from “Small” Shape.

These results reveal that the maximum principal stress in the “weak zone” is highly influenced by the initial cervical shape and almost independent of cervical stiffness.

### Cervical contraction

**Fig. 20:**
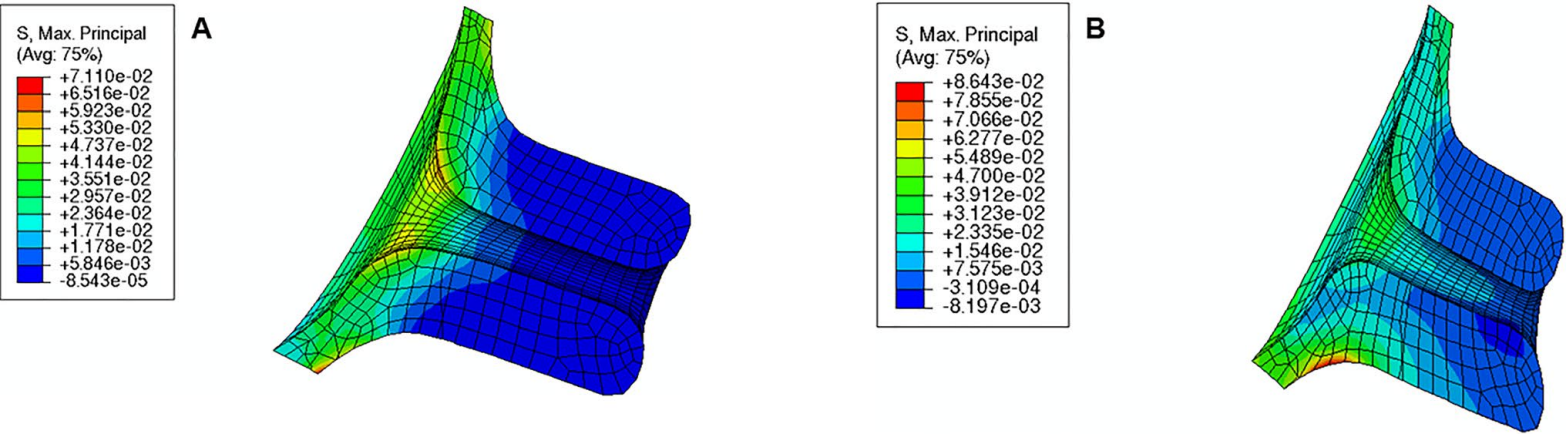
Maximum principal stress distribution for “Large” shape: **A** passive soft cervix, **B** active soft cervix (units: MPa)

**Fig. 21:**
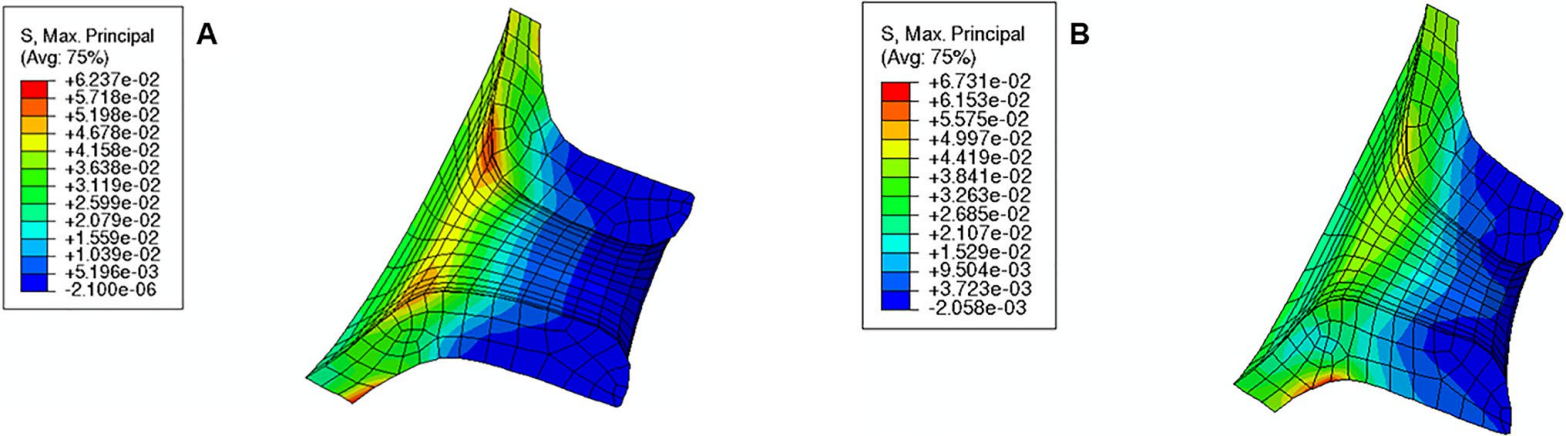
Maximum principal stress distribution for “Mid” shape: **A** passive soft cervix, **B** active soft cervix (units: MPa)

**Fig. 22:**
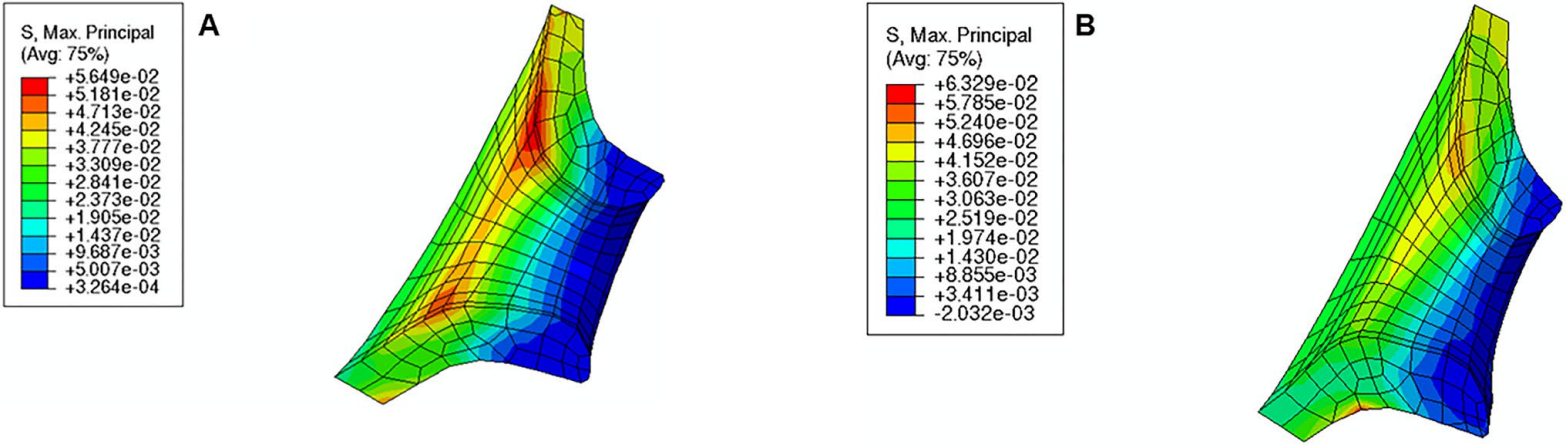
Maximum principal stress distribution for “Small” shape: **A** passive soft cervix, **B** active soft cervix (units: MPa)

The maximum principal stress values and distributions caused by cervical contractions are illustrated in Figs. 20, 21, and 22. First, the maximum principal stress values increase when cervical contractions are activated, independently of the initial cervical shape. Second, the highest stress values are extended to the cervical canal and not restricted to the internal *os* region, as observed in previous results. Finally, the cervices deform differently compared to the respective passive cervix.

**Fig. 23:**
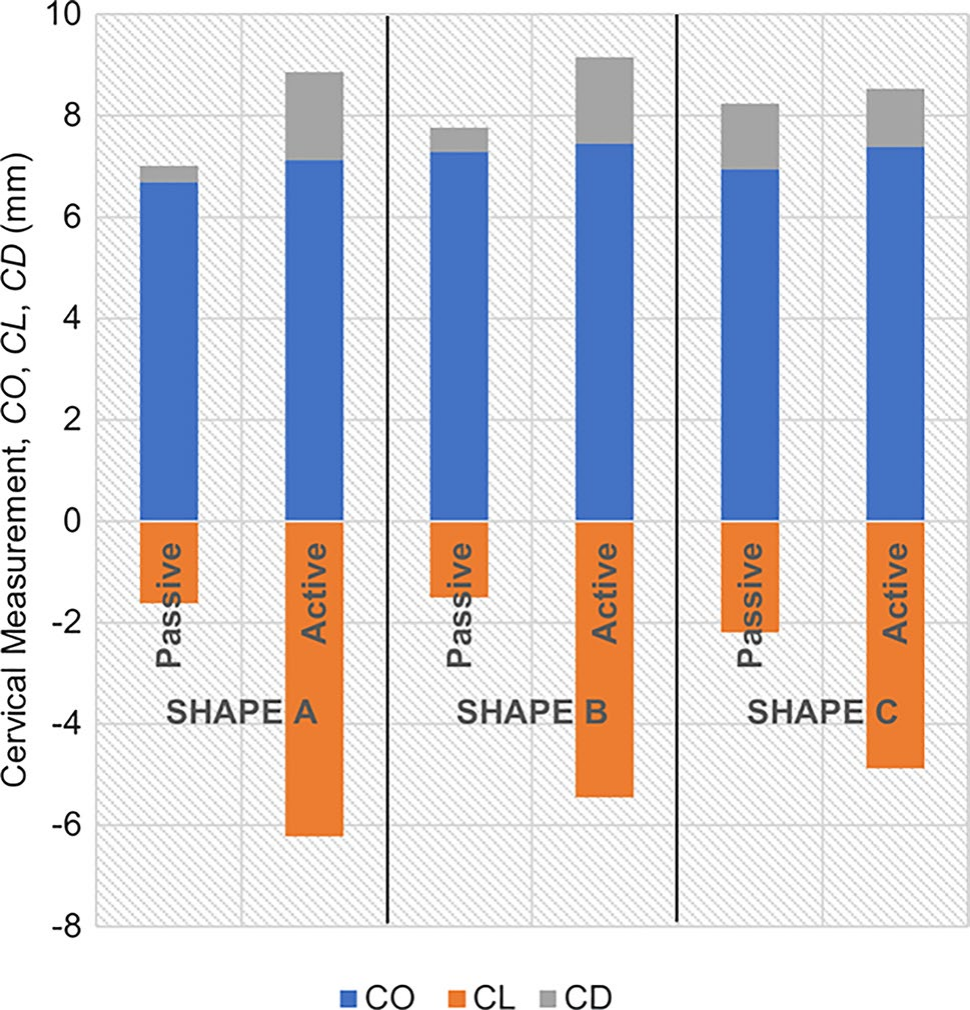
Variations of cervical opening (CO), cervical length (CL), and cervical dilation (CD) for different cervical shapes. “Large” shape, “Mid” shape, and “Small” shape represent different cervical shapes as described in Sect. 2.3, while “passive” and “active” represent a soft cervix with no contraction and a soft cervix contracting, respectively

From the analysis of Fig. 23, it is possible to affirm for all cervical shapes that active cervical contractions generally increase the absolute values of CO, CL, and CD compared to those from the passive soft cervix. CL is largely affected by cervical contractions, followed by CD and CO. As the cervix gets shorter and more dilated, the impact of cervical contraction on those measurements tends to get less significant.

The cervical contractions do not interfere significantly with the maximum stress values verified at the “weak zone” of the amnion layer, compared to the passive soft cervices analyzed in subSect. 3.2.

### Intrauterine pressure

**Fig. 24:**
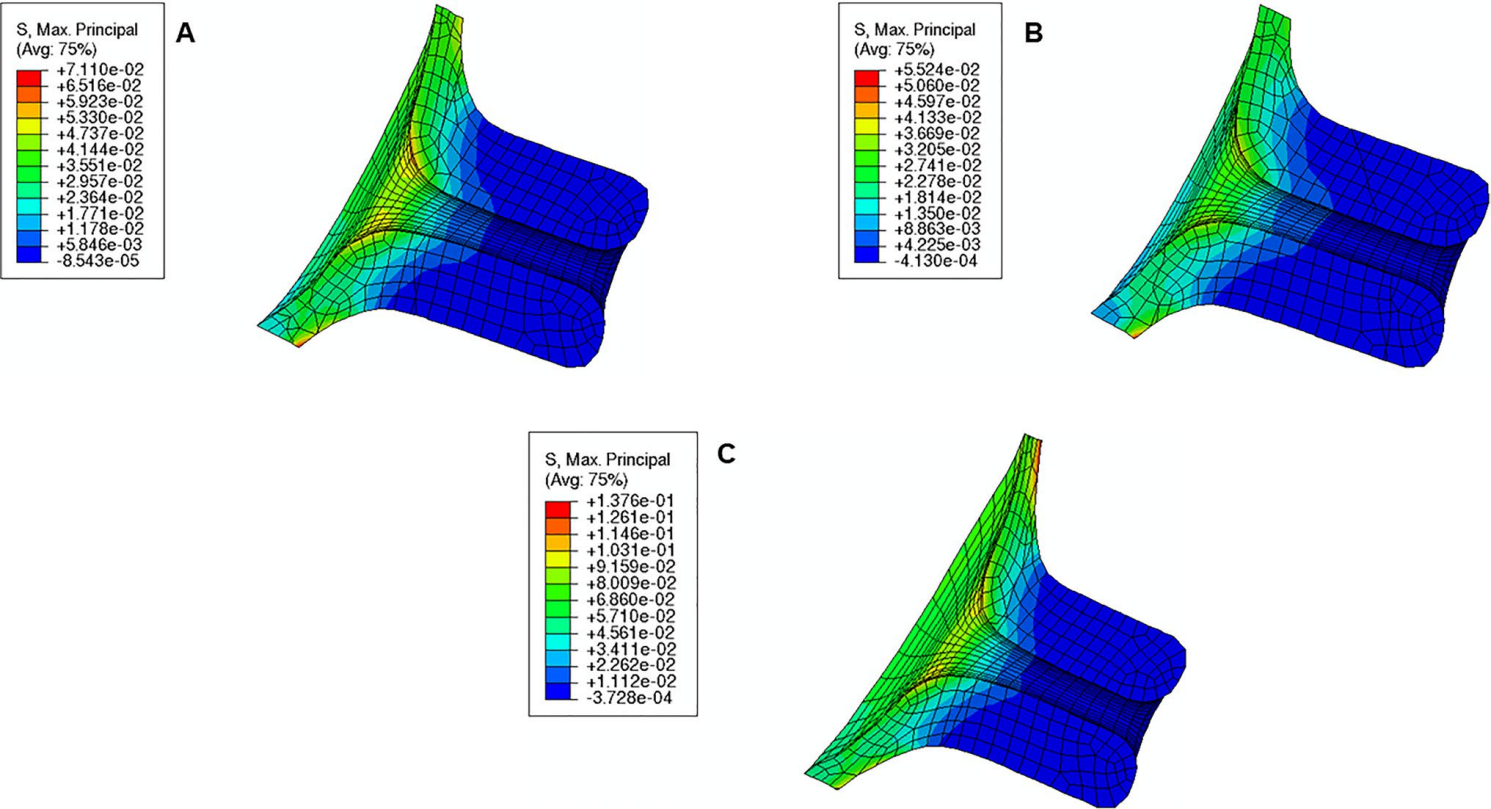
Maximum principal distribution in the cervix for different intrauterine pressures (IUP) values: **A** normal IUP, **B** smaller IUP, **C** larger IUP (units: MPa). These results were retrieved using the soft cervix from “Large” shape

The maximum principal stress values and distributions caused by different intrauterine pressures (IUP) are illustrated in Fig. 24. As expected, increasing the IUP leads to increased stresses within the cervix, where the internal *os* region is subjected to larger stresses.

IUP largely influences CO, which experiences a large increase when IUP is elevated and a significant decrease when IUP is also decreased (Table 6). CL also experiences a similar evolution despite being less impactful. A smaller IUP does not largely influence CD, but its value increases when the pressure is elevated.

**Table 6:** Variations of cervical opening (CO), cervical length (CL), and cervical dilation (CD) for different intrauterine pressures (IUP)

	“Large” shape		
	Smaller IUP	Normal IUP	Larger IUP
CO (mm)	3.736	6.689	14.752
CL (mm)	− 1.239	− 1.619	− 2.520
CD (mm)	0.363	0.312	0.631

“Large” shape represents the longest and most closed cervix as described in Sect. 2.3

Finally, in terms of maximum principal stress at the “weak zone” of the amnion layer, the following values are retrieved for the soft cervix from “Large” Shape:

- 0.3 MPa for the normal IUP;
- 0.2 MPa for the smaller IUP;
- 2.0 MPa for the largest IUP.

These results reveal that the maximum principal stress in the “weak zone” is largely influenced by the IUP.

## Discussion

This work represents the first computational investigation of the mechanical interaction between the cervix, fetal membrane, and uterine body before the onset of labor, employing uterine and cervical contractions and various biological features, such as intrauterine pressure, amniotic fluid, and a multilayer fetal membrane. The impact of (i) initial cervical shape, (ii) cervical stiffness, (iii) cervical contractions, and (iv) intrauterine pressure was investigated on the cervix and fetal membrane in terms of maximum principal stress and cervical shape change.

Uterine contractions influence cervical remodeling in terms of shape before the onset of labor: The cervix can only dilate sufficiently upon the initiation of uterine contractions (Timmons et al. 2010). Thus, these contractions were added to the uterine model. In our work, the maximum principal stress value in the uterine muscle was 0.091 MPa (Fig. 14), and the stress values obtained were within the physiological range of a pregnant human uterine body, as reported by Blisplinghoff et al. (Bisplinghoff et al. 2012).

A multilayer fetal membrane was integrated into the gravid uterine model, comprising the amnion, chorion, and part of the decidua. The amnion was subjected to much larger maximum principal stresses than the chorion Fig. 15. Experimental studies show that the amnion is the fetal membrane’s mechanically dominant layer, withstanding most loads occurring during pregnancy (Buerzle and Mazza 2013). Moreover, the stress values for both layers share the same magnitude as other results from the literature (Verbruggen et al. 2017).

During pregnancy, the cervix goes from a closed structure to one that is soft and compliant (Kitamura et al. 2023; Myers et al. 2015; Timmons et al. 2010). It must open sufficiently for birth before the onset of labor through a remodeling process that will lead to cervical dilation and shortening (Timmons et al. 2010). Cervical insufficiency occurs when the cervix is weak and unable to remain closed until late delivery, leading to preterm birth (Wierzchowska‐Opoka et al. 2021). On the other hand, a closed stiff cervix may create complications during cervical dilation and lead to failed induced labor (Mazza et al. 2014). These phenomena highlight the importance of cervical shape and stiffness to a successful vaginal delivery. According to Figs. 16, 17, and 18, maximum principal stress values decreased as the cervix got more dilated, shorter, and softer. Larger stresses were found in the internal os region of the cervix, where the fetal membrane was allowed to slide freely. This observation agrees with the results obtained by Paskaleva et al., where it was demonstrated that loads were concentrated in the internal os (Anastassia Paskaleva 2007). Moreover, as cervical stiffness got smaller, cervical dilation and opening increased, and cervical length decreased, independently of the initial cervical shape (Fig. 19).

A growing body of work exists on the passive behavior of the cervix, but understanding the role of the cervical smooth muscles during pregnancy has been neglected (Tantengco and Menon 2020). It has been hypothesized that the contraction and relaxation of the cervical smooth muscle may be involved in cervical remodeling, preparing the cervix for labor (Tantengco and Menon 2020). In this work, we investigated the impact of cervical contractions in the longitudinal direction on cervical dilation and shortening. First, the cervical contractions increased the maximum principal stress values and changed the stress distributions, interfering with the cervical deformation before labor (Figs. 20, 21, and 22). Additionally, the cervical contraction potentiated cervical shortening and dilation, leaving the cervix more prepared for labor than the respective passive cervix (Fig. 23). These results highlight the importance of cervical contractions for cervical remodeling and may offer solutions to some complications at the initial stages of vaginal delivery, such as a failed induced labor: To proceed to induction, the cervix must be dilated and short enough to use oxytocin (Harper et al. 2012); if the cervix is not prepared, some medical alternatives are available, such as cervical ripening; however, none of the methods for cervical ripening tries to stimulate cervical contractions to prepare the cervix for labor.

Intrauterine pressure (IUP) is elevated before the onset of labor due to uterine contractions (Smith 1984). It will push the amniotic sac in the region of the cervical opening against the relaxed cervix, starting the gradual dilation (Smith 1984). This process was verified in Fig. 24 and Table 6, where larger IUP led to considerably larger stresses and cervical openings, larger dilation, and smaller cervical lengths. In clinical practice, if a small IUP is registered, cervical remodeling may be too slow. Inducing uterine contractions may elevate IUP and speed up the cervical remodeling process before labor, as shown in this work.

The rupture of human fetal membranes occurs in the cervical opening region called the “weak zone” (El Khwad et al. 2005). Biaxial strength, defined as the maximum stress at failure, was calculated experimentally through a puncture mechanical test (Oyen et al. 2004). For fetal membrane samples from labored term pregnancies, it was found that the minimum biaxial strength value was approximately 0.6 MPa (Oyen et al. 2004). According to this work, this value was only surpassed for the shortest and more dilated cervix, illustrated in Fig. 18, where a value of 0.7/0.8 MPa was verified at the “weak zone,” and for a larger IUP, illustrated in Fig. 24C, where a value of 2.0 MPa was observed at the same region. These results highlight the important interaction between fetal membrane rupture, cervical geometry, and IUP, and may explain the preterm premature rupture of the fetal membrane (PPROM) in spontaneous preterm birth: in this case, cervical softening and opening, as well as uterine contractions, are verified too soon (Mercer 2010); the premature remodeling and larger IUP caused by uterine contractions may be the cause of PPROM since those factor potentiate fetal membrane rupture according to our results.

Some simplifications were considered in this work: (i) the nonlinear behavior of the chorion and the decidua was not considered in the layered fetal membrane model, since it is particularly difficult to isolate these layers for experimental testing, (ii) the poro-viscoelastic behavior of the uterus/cervix was not considered, (iii) the reference configuration is already a deformed state, (iv) collagen fiber dispersion in the uterus/cervix was neglected, and (v) only one contraction was simulated, due to computational cost.

## Conclusion

This work investigated the impact of (i) initial cervical shape, (ii) cervical stiffness, (iii) cervical contractions, and (iv) intrauterine pressure on cervical shape change and membrane rupture, highlighting the important interaction between the cervix, fetal membrane, and uterine contractions. Relevant mechanical conclusions were retrieved from this work, reinforcing the importance of understanding this complex interaction to guide the clinical approach to several complications, such as failed induced labor and preterm birth.
